# Development and validation of matrix-validated LC–MS/MS method for simultaneous quantification of 21 neonicotinoids and their metabolites in human urine

**DOI:** 10.1007/s11356-026-37779-9

**Published:** 2026-05-01

**Authors:** Wenjing Xi, Sarah Onysio, Mary C. Rhodes, Alexis J. Faudel, John C. Flunker, Mark K. Santillan, Donna A. Santillan, David M. Cwiertny, Darrin A. Thompson

**Affiliations:** 1 Center for Health Effects of Environmental Contamination, University of Iowa, Iowa City, IA USA; 2 Department of Occupational and Environmental Health, College of Public Health, University of Iowa, Iowa City, IA USA; 3 Department of Obstetrics and Gynecology, University of Iowa, Iowa City, IA USA; 4 Department of Epidemiology, College of Public Health, University of Iowa, Iowa City, IA USA; 5 Department of Civil & Environmental Engineering, University of Iowa, Iowa City, IA USA; 6 Department of Chemistry, University of Iowa, Iowa City, IA USA

**Keywords:** Neonicotinoid insecticides, Clothianidin-n-desmethyl, Imidacloprid olefin, Thiamethoxam urea, LC–MS/MS, Urine, Human exposure assessment risk

## Abstract

Neonicotinoids (NEOs) are the most widely used class of insecticides worldwide. Consequently, human exposure occurs through multiple pathways, including dietary intake and environmental contact. Previous analytical methods have primarily focused on parent compounds, often excluding key metabolites due to matrix effects and poor quantification in complex biological samples. This gap severely limits the ability to accurately assess total NEO exposure and internal dose, highlighting the urgent need for more comprehensive and reliable biomonitoring approaches. This study developed a comprehensive LC–MS/MS method to quantify 21 NEOs and metabolites in human urine, uniquely including key transformation products such as clothianidin-n-desmethyl, clothianidin-urea, imidacloprid-urea, imidacloprid-olefin, 5-hydroxy-imidacloprid, thiamethoxam-urea, and thiamethoxam-n-desmethyl, thereby enabling a more thorough assessment of human exposure to NEOs. The method was validated using both synthetic and pooled human urine, providing a critical assessment of matrix-dependent variations in detection limits, accuracy, and precision. Method performance demonstrated high sensitivity, specificity, accuracy, and precision, with limits of quantification (LOQ) from 0.017 to 0.46 ng/mL and limits of detection (LOD) from 0.0050 to 0.14 ng/mL. The method was applied to urine samples from 246 pregnant women and 47 farmers in eastern Iowa to assess exposure levels. Results indicated widespread NEO exposure with detection frequencies of 99% of pregnant women and 100% in farmers, with 18 different NEOs and metabolites detected across both populations. Metabolite monitoring revealed that imidacloprid metabolites (IMI-O, 5-OH-IMI) were stronger predictors of total neonicotinoid burden than parent compounds alone (*r*_s_ = 0.735 vs < 0.6 for parents), with some metabolites showing higher concentrations in pregnant women than farmers despite farmers having higher overall exposure. These findings demonstrate that parent-only biomonitoring substantially underestimates internal neonicotinoid exposure and fails to capture critical exposure pathways, particularly for vulnerable populations where metabolite patterns may differ significantly from occupational exposure profiles.

**Supplementary Information:**

The online version contains supplementary material available at 10.1007/s11356-026-37779-9.

## Introduction

Human exposure to neonicotinoids (NEOs) and their metabolites is widespread, with biomonitoring studies documenting their presence in populations all over the world (Baker et al. 2019; Mahai et al. 2023; Ospina et al. 2019; Pan et al. 2023, Zhang & Lu 2022). This ubiquity is driven in part by the physicochemical properties of NEOs: they display high water solubility (Terayama et al. 2022), have relatively long environmental half-lives, and exhibit low sorption in soil (Hladik et al. 2017; Thompson et al. 2020). As a result, NEOs are frequently detected in surface and groundwater (Berens et al. 2020; Hayashi et al. 2021; Wan et al. 2021), soil (Morrissey et al. 2015; Stewart et al. 2014), household dust (Bennett et al. 2019), and air (Tapparo et al. 2012). For most individuals, ingestion of food (Chen et al. 2014) and drinking water (Berens et al. 2020; Bradford et al. 2018; Sultana et al. 2018; Thompson et al. 2023; Wan et al. 2020) are believed to be the primary routes of exposure. Exposure can also occur in occupational settings during agricultural application (Lee et al. 2022; Nishizawa et al. 2022; Suwannarin et al. 2021) or residential pesticide use (Thompson et al. 2020).

In humans, NEOs are metabolized and excreted efficiently due to their high water solubility and low lipophilicity (Thompson et al. 2020). Some NEOs, including clothianidin (CLO), dinotefuran (DIN), and nitenpyram (NIT), are primarily eliminated in urine unchanged (Li & Kannan 2020, Tomizawa & Casida 2005). However, most NEOs undergo biotransformation through hydroxylation, dehydrogenation, or nitroimine reduction (Li et al. 2020; Wang et al. 2018). Some of these derivatives, such as N-unsubstituted amines, have stronger binding affinity for mammalian nicotinic acetylcholine receptors (Tomizawa et al. 2000) and exhibit greater toxicity than their parent compounds (Dai et al. 2006, Ford 2008, Ford & Casida 2006, la Farre et al. 2008, Nauen et al. 2001). These metabolic characteristics highlight the importance of monitoring both parent NEOs and their biologically relevant transformation products in human biomonitoring studies.

In an Iowa study published in 2023, our group documented NEO exposure in every sample collected from a small cohort of farmers (Thompson et al. 2023). Although the analytical method quantified 14 analytes, only a limited number of metabolites specific to clothianidin (CLO), imidacloprid (IMI), and thiamethoxam (THX)—three NEOs heavily used in the region—were included. Despite detecting these parent NEOs in more than half of the participants’ drinking water samples, the study concluded that water was unlikely to be a major exposure source and suggested that diet may instead be a more prominent contributor. In a subsequent environmental monitoring study, our research group identified several distinct metabolites for these NEOs occurring in water, including clothianidin-n-desmethyl (CLO-N-DES), clothianidin-urea (CLO-U), imidacloprid-urea (IMI-U), imidacloprid-olefin (IMI-O), and thiamethoxam-urea (THX-U) (Xi et al. 2024). The presence of these metabolites in the environment indicated that earlier biomonitoring approaches may have overlooked an important portion of total NEO exposure, underscoring the need for a broader and more targeted analytical method to better understand the extent of human exposure from environmental sources. This need is particularly pressing in Iowa, where environmental exposures are of increasing concern amid rising cancer incidence rates.

To address these gaps, the present study had three primary objectives: (1) to develop and analytically validate a comprehensive urinary method capable of quantifying an expanded panel of 21 NEOs and metabolites; (2) to evaluate potential matrix effects and performance characteristics relevant to population-based biomonitoring; and (3) to apply this method to human samples to further validate method performance.

Researchers developed and validated a quantitative method enabling simultaneous measurement of 21 analytes through a single urinary assessment. This expanded panel includes key metabolites—such as CLO-N-DES, CLO-U, IMI-U, IMI-O, 5-hydroxy-imidacloprid (5-OH-IMI), THX-U, and N-desmethyl thiamethoxam (THX-N-DES)—that reflect environmental transformation products and may serve as sensitive biomarkers of exposure in populations residing in regions of intensive NEO use (US Geological Survey, 2018). The method was intentionally designed as a companion to a previously published environmental water method (Xi et al. 2024), allowing for more integrated evaluation of potential exposure sources.

This urine method was used to assess NEO exposure among 246 pregnant women who provided samples between 2010 and 2024 through the University of Iowa’s Perinatal Family Tissue Bank (PFTB) (Santillan et al. 2014). A secondary analysis was also conducted on stored samples from the 47 farmers considered in our earlier study (Thompson et al. 2023) to focus on metabolites that had not been previously quantified in that population. By comparing exposures between farmers—who represent a high-exposure group due to occupational pesticide use—and pregnant women—a vulnerable population because of potential maternal and fetal health implications—this study aims to provide context for future research examining health risks and exposure pathways.

## Materials and methods

### Reagents and chemicals

High-performance liquid chromatography (HPLC)-grade methanol, acetonitrile, and ethyl acetate, as well as Optima™ LC/MS-grade formic acid, were obtained from Fisher Scientific (Pittsburgh, PA, USA). Ultrapure water was produced in-house using a Milli-Q purification system (MilliporeSigma, Bedford, MA, USA). Urine samples were analyzed for ten NEOs and eleven metabolites. 100 mg/mL of ACE, CLO, IMI, THC, THX, NIT, flupyradifurone (FLU), flonicamid (FLO), sulfoxaflor (SUL), and THX-N-DES in methanol or in acetonitrile were purchased from Chem Service (West Chester, Pennsylvania, USA). Imidaclothiz (IMZ) was purchased from Santa Cruz Biotechnology (Dallas, Texas, USA). THC amide (THC-A) was obtained from Sigma-Aldrich (St. Louis, Missouri, USA). 10 mg/mL of IMI-U, and 100 mg/mL of acetamiprid-n-desmethyl (ACE-N-DES), CLO-U, and IMI-O, *D*3-ACE, *D*3-CLO, *D*4-imidicloprid, *D*4-THC, *D*3-THX, and *D*3-flonicamid in acetonitrile or acetone, and solid powders of CLO-N-DES, THX-U, NIT-N-DES, and sulfoxaflor MTB X11719474 (SUL-X) were purchased from HPC Standards Inc. (Atlanta, Georgia, USA). 100 mg/mL of 5-OH-IMI, isotopically labeled 5-OH-IMI (2-^13^C, 3-^15^N, 2-amino-^15^N), THC-A (pyridylmethyl-^13^C6), CLO-N-DES (Guanidine-^13^C, Guanidine-1,3-^15^N2), and IMI-O (Imidazol-1-^15^N, 2-^13^C, 2-amino ^15^N) were purchased from Cambridge Isotope Laboratories (Tewksbury, Massachusetts, USA). 3-^2^H,^13^C, 2-^15^N-ACE-N-DES was purchased from CanSyn Chemical Corporation (Toronto, Ontario, Canada). *D*3-NIT was ordered from C/D/N Isotopes (Pointe-Claire, Quebec, Canada). *D*4-imidaclothiz was purchased from Toronto Research Chemicals Inc (Toronto, Ontario, Canada). All isotope-labeled compounds were used as internal standards (ISTDs). Manufacturing, CAS number, and molecular weight (MW) for all target analytes and their assigned isotope-labeled internal standards are listed in Table S1. Synthetic urine and pooled urine were purchased from Sigma-Aldrich (St. Louis, Missouri, USA). Urine samples collected from pregnant women were measured for creatinine by the Center for Health Effects of Environmental Contamination and the PFTB (Bioassay Systems Quantichrom Creatinine Assay Kit).

### Study participants and sampling

Urine samples were collected from two sources. First, 246 samples collected by the PFTB (Santillan et al. 2014) between 2010 and 2025 from pregnant women were analyzed. Anonymized samples provide alongside with only the dates of sample collection and pregnancy, were provided by PFTB to the University of Iowa’s Center for Health Effects of Environmental Contamination (CHEEC) to assist with method development and to provide a historical snapshot of past exposures. Next, samples from 47 farmers that were adult male pesticide applicators were retested for the metabolites of CLO, IMI, and THX. Details on the collection and prior results have been previously published (Thompson et al. 2023). These samples were originally analyzed for fourteen analytes, including ACE, CLO, DIN, FLO, IMI, IMZ, NIT, SUL, THC, THX, 6-CN, ACE-N-DES, THC-A, and THX-N-DES. The parent FLU and the metabolites CLO-N-DES, CLO-U, 5-OH-IMI, IMI-U, IMI-O, NIT-N-DES, SUL-X, and THX-U were not measured as part of this earlier study. First-morning-void samples from this prior study were re-tested to validate this new method and to examine potential differences in exposure between male farmers and pregnant women in Iowa.

This study used pre-existing, IRB-approved biorepository samples. Human subjects research approval was provided by the IRB at the University of Iowa for both studies IRB# 200901784, 201809848, and 201902830, respectively. All samples were stored at −80 °C after collection until analyzed following the US Center for Disease Control and Prevention’s guidance for long-term storage of non-persistent pesticides (Division of Laboratory Sciences, National Center for Environmental Health, Centers for Disease Control and Prevention 2018).

### Preparation of standard

Individual stock solutions (1 mg/mL) for solid standards were first weighed individually and then dissolved in acetonitrile (ACN). A mixed working stock (1000 ng/mL) was then prepared by combining aliquots of the individual analyte solutions and adjusting the final volume with acetonitrile (ACN). Mixed stocks were then serially diluted with ACN to yield concentrations of 100, 10, and 1 ng/mL for spiking solutions. These solutions were renewed every three months. All standards were stored below −10 °C.

Stable isotopically labeled internal standards (ISTDs) obtained in solid form were dissolved in ACN at 0.2–0.5 mg/mL to prepare individual ISTD stocks. A composite ISTD stock (1000 ng/mL) was prepared by mixing individual solutions and diluting with ACN. The ISTD working solution (100 ng/mL) used for sample fortification was prepared by further dilution of the mixed stock.

Calibration standards (1 mL each) were freshly prepared before each analytical batch by spiking appropriate amounts of analyte working solutions into 0.25% formic acid in ACN/Milli-Q water (1:9, v/v), covering a concentration range of 0.01–25 ng/mL. Each calibrator also received 50 µL of the 100 ng/mL ISTD working solution, resulting in a final ISTD concentration of 5 ng/mL. The concentrations and volumes used for the preparation of all calibration standards are listed in Table S2.

### Quality controls

During method development and validation, quality controls (QC) were generated using both synthetic and pooled human urine matrices. To prepare the QC materials, 1 mL aliquots of urine were fortified with native analytes to achieve concentrations corresponding to QC Low (QCL) (0.025 ng/mL), QC Medium Low (QCML) (0.25 ng/mL), QC Medium High (QCMH) (1.5 ng/mL), and QC High (QCH) (4.5 ng/mL). Details of the preparation scheme are summarized in Table S2. For ongoing sample analysis, routine QC materials were produced from pooled urine. The pooled urine was subdivided into three 50 mL portions, and each was spiked to generate low (≈ 0.35 ng/mL), medium (≈ 1.75 ng/mL), and high (≈ 4.75 ng/mL) QC levels. After homogenization, the material was aliquoted into 1.2 mL polypropylene vials and stored at  −80 °C until use.

### Extraction of QCs and unknown samples

Prior to extraction, frozen QC and unknown samples were thawed, centrifuged, and 1 mL of the clarified supernatant was transferred to a 16 × 100 mm borosilicate glass tube. Samples were briefly vortex-mixed at room temperature, followed by the addition of 10 mL of Milli-Q water and 25 µL of 100 ng/mL internal-standard (ISTD) solution (see Table S2). The ISTD was added prior to SPE so that it underwent the same extraction, cleanup, and ionization processes as the analytes, allowing correction for extraction recovery and matrix effects. The mixture was vortexed for 1 min before solid-phase extraction (SPE). Automated extractions were carried out on a Vivace™ Duo Cleanup Station (PromoChrom Technologies, Richmond, BC, Canada) using Strata™-X-CW polymeric weak-cation cartridges (60 mg/3 mL; 33 µm; Phenomenex, Torrance, CA, USA). The detailed conditioning, washing, drying, and elution steps are summarized in Table S3. Specifically, cartridges were conditioned sequentially with methanol followed by Milli-Q water prior to sample loading. After the diluted sample was loaded, cartridges were washed with water and 20% methanol in water to remove salts and polar matrix components, followed by air and nitrogen drying to eliminate residual moisture. Analytes were then eluted using 0.5% formic acid in acetonitrile/ethyl acetate (8:2, v/v). The final extracts were evaporated to dryness, reconstituted in 0.25% formic acid in ACN/Milli-Q water (1:9, v/v), and transferred into LC vials for analysis.

### Instrument analysis

The HPLC-tandem mass spectrometry system used was an Agilent 1290 Infinity LC (Agilent Technologies, Santa Clara, CA, USA) coupled to an AB Sciex 6500 plus triple quadrupole mass spectrometer (Framingham, MA, USA) equipped with a Kinetex Biphenyl column (2.1 × 100 mm, 5 µm; Phenomenex, Torrance, CA, USA). Two mobile phases were used—(A) 0.1% formic acid in Milli-Q water and (B) 0.1% formic acid in acetonitrile. The flow rate, gradient program, MS/MS parameters and transitions used were identical to those in a previously published method (Xi et al. 2024). Representative chromatograms of analytes spiked in solvent and extracted pooled urine are shown in Figure S1.

### Optimization of sample preparation

Three types of solid-phase extraction (SPE) cartridges: Oasis HLB (3 mL/60 mg, 30 μm; Part No: WAT094226, Waters, Milford, MA, USA), Bond Elut Plexa (3 mL/60 mg, 45 μm; Part No: 12108603 Phenomenex), and Strata™-X-CW were tested with 10% methanol in water (v/v) as the washing solvent to find optimal extraction conditions. Next, 10–30% of methanol in water (v/v) was investigated as washing solvent during extraction with Strata™-X-CW as the SPE cartridge. To prepare the matrix spike, 1 mL of pooled urine was fortified with the stock solution to achieve an analyte concentration of 1.5 ng/mL. The spiked sample was processed according to the procedure outlined in the “Quality controls” section. Subsequently, the extracts were analyzed, and the average peak area for each analyte was used to calculate process efficiency (PE), following the approach described in previous studies (Cortese et al. 2020; Xi et al. 2024). Specifically, PE was calculated by comparing the analyte peak area obtained from matrix samples fortified prior to extraction with the peak area of standards prepared in solvent at the same nominal concentration:

PE (%) = (A_pre-extraction_/A_neat_) × 100 (1)

where A_pre-extraction_ is the average peak area measured in samples spiked before extraction and A_neat_ represents the average peak area from neat standard solutions at the same analyte concentration.

### Method validation

Linearity, sensitivity, specificity, accuracy, and precision were used to assess overall method performance. All validation experiments, including calibration modeling, determination of limits of detection (LOD) and quantification (LOQ), and assessments of selectivity, accuracy, and precision were conducted following previously established procedures (Xi et al. 2024). To evaluate matrix-related effects on method performance, QC samples were prepared in both synthetic urine and pooled urine at five levels (matrix blank, QCL, QCML, QCMH, and QCH), with nine independent replicates at each level. The samples were analyzed on different days.

Limits of detection (LOD) and limits of quantification (LOQ) were estimated from low-level fortified quality control samples (QCL). Nine QCL samples were independently prepared, extracted, and analyzed on different days. Each extract was injected three times, and the measured concentrations from all injections were used to estimate the standard deviation (σ) of the method response. LOD and LOQ were calculated as 3σ and 10σ, respectively. For most analytes, σ was estimated using low-level fortified pooled urine. However, for analytes exhibiting measurable endogenous concentrations (i.e., neonicotinoids naturally present in the sample prior to analysis) in pooled urine blanks, the background signal interfered with estimation of variability at low added levels. In these cases, low-level fortified synthetic urine was used to determine σ while maintaining the same extraction and analytical procedure. This approach allowed estimation of detection capability without bias from pre-existing analyte concentrations in pooled urine. LOD and LOQ values determined in synthetic urine are referred to as method detection limits, whereas values obtained in pooled urine are reported as practical reporting limits due to endogenous background.

Matrix effect (ME) was evaluated by comparing the analyte peak area obtained from matrix extracts fortified after extraction with the peak area of standards prepared in solvent at the same nominal concentration:

ME (%) = [(A_post-extraction/A_neat) − 1] × 100 (2)

where A_post-extraction represents the peak area from matrix samples spiked after extraction and A_neat represents the peak area from neat standard solutions. Negative values indicate ion suppression and positive values indicate ion enhancement.

Because endogenous levels of several target analytes were detected in the pooled urine blank, the mean concentrations measured in blank samples were first determined and subsequently used to calculate blank-corrected recoveries according to Eq. (2) (Cortese et al. 2020):

Blank corrected recovery% = 100 × $\frac{\text{(final concentration - initial concentration)}}{\text{spiked concentration}}$% (3)

For QC samples prepared in synthetic urine, accuracy was expressed as the ratio of the measured concentration to the spiking level, while precision was reported as the relative standard deviation (RSD, %) of the measured concentrations.

### Data analysis

Raw data were processed and integrated using SCIEX OS software (version 3.3.1.43; AB Sciex, Framingham, MA, USA), and quantification was based on peak area responses. Each analytical batch typically began with three injections of a mid-range calibrator to equilibrate the system, followed by a solvent blank to monitor potential carry-over, a laboratory reagent blank containing internal standards (LRB), 10–12 calibration standards, a water rinse, three matrix spikes prepared with pooled urine (QCL, QCM, and QCH), one Milli-Q water extract, and up to 150 processed urine samples per LC–MS/MS run. One calibration standard was re-analyzed after every ten samples to ensure that instrument response remained stable throughout the run. The detection frequency (DF) for each analyte was defined as the proportion of samples with concentrations exceeding the limit of detection (LOD). For detections between the LOD and the limit of quantification (LOQ), values equal to one-half of the LOQ were used in subsequent calculations. Non-detects were assigned a value equal to half the detection limit, based on the assumption that this substitution would minimize bias and provide a more accurate estimate of the mean (EPA 2017).

The distribution of continuous variables and their natural log-transformed values was evaluated for normality using the Shapiro-Wilk test. Because several analytes were skewed, descriptive statistics—including the median, quartiles, and maximum—are reported. Associations between continuous variables were evaluated using Spearman’s rank correlation. Univariate analyses were conducted using nonparametric tests (Wilcoxon Rank Sum or Kruskal-Wallis) to compare NEO concentrations across available metadata categories.

To evaluate potential changes in exposure over time, descriptive statistics and temporal trends for all analytes were examined using annual, 2-year, and 5-year groupings across the 2010–2025 collection period. These groupings were initially assessed to determine the most appropriate temporal resolution that balanced statistical power, stability of estimates, and interpretability of trends. Annual comparisons were limited by small sample sizes, with some years containing as few as nine specimens, which reduced statistical power and increased year-to-year variability. Two-year intervals provided a better balance between sample size and temporal resolution. Each 2-year period contained at least 25 samples, resulting in more stable and reliable estimates. Five-year intervals provided larger sample sizes but lacked sufficient temporal granularity to detect more subtle trends. Full comparisons across annual, 2-year, and 5-year intervals are provided in the Supplemental Information (Tables S4 and S5), while the main text primarily describes results using 2-year structures.

Aggregate metrics were also assessed because parent NEOs share parent-metabolite relationships and are shown to co-occur. These totals were calculated to provide an indicator of exposure burden (Table 1). ƩIMI represents the sum of concentrations from IMI, 5-OH-IMI, IMI-O, and IMI-U. ƩCLO is equivalent to the sum of CLO, CLO-U, and CLO-N-DES. ƩACE is ACE plus the concentration of ACE-N-DES. Since CLO is a metabolite of THX, ƩTHX includes Total CLO and the sum of THX, THX-U, and THX-N-DES. The sum of all analytes is labeled as ƩNEO.

**Table 1:** Definition for aggregate metrics used to assess parent-metabolite co-occurrence

Aggregate metric	Analytes included
ƩNEO	Sum of all NEOs concentrations measured
ƩACE	ACE + ACE-N-DES
ƩCLO	CLO + CLO-U + CLO-N-DES
ƩIMI	IMI + 5-OH-IMI + IMI-O + IMI-U
ƩTHX	THX + N-DES-THX + THX-U + CLO + CLO-U + CLO-N-DES

Creatinine measurements for farmers were previously completed by the National Center for Environmental Health, US Centers for Disease Control and Prevention (Thompson et al. 2023). NEO concentrations have been adjusted for creatinine to normalize urinary concentrations of analytes and reported as μg/g.

SAS 9.4 (SAS Institute, Cary, NC) was used to perform most of the statistical analyses. Descriptive statistics were calculated to summarize neonicotinoid concentrations.

## Results and discussion

### Method development, optimization, and validation

#### Extraction method optimization

This method was developed following the same steps and processes described previously in a published method for water (Xi et al. 2024). with optimization for the cartridge and washing solvent. Three cartridges, Oasis HLB, Bond Elut Plexa, and Strata™-X-CW, were chosen in this study. The Oasis HLB SPE cartridge is commonly used to extract NEOs from water (Wan et al. 2020, 2019; Webb et al. 2021) and urine (Gao et al. 2022; Wang et al. 2020; Zhou et al. 2021), while the Bond Elut Plexa cartridge from Phenomenex is also widely applied for urine extraction (Honda et al. 2019, Li & Kannan 2020). In addition, Strata™-X-CW was found to provide good recoveries for both NEOs and their metabolites in other matrices (Gbylik-Sikorska et al. 2015). The process efficiency (PE) described in the “Instrument analysis” section was then calculated and compared for each analyte.

**Fig. 1:**
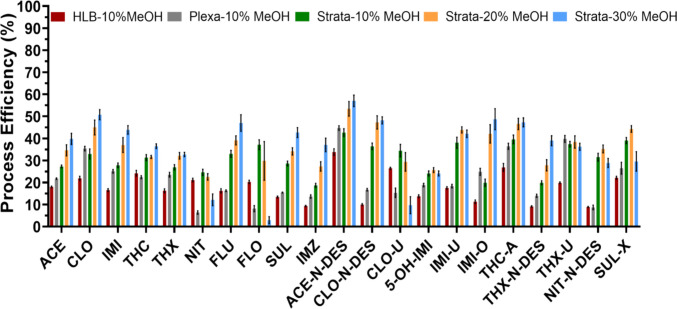
Process efficiency (%) of neonicotinoids and their metabolites in pooled urine (1.5 ng/mL) using three solid-phase extraction cartridges: HLB (Waters), Plexa (Phenomenex), and Strata-X-CW (Phenomenex). For Strata-X-CW, data are shown with three different washing solvents: 10%, 20%, and 30% methanol in water (v/v, 100%). For all analyses, *n* = 9; three samples were prepared at each condition and then three injections were made for each sample. Error bars represent the standard deviation

PE was measured under different SPE conditions for each analyte. The PE is presented in Fig. 1. PE values for most analytes ranged from approximately 20 to 60% across the various washing solvents and cartridge types tested. Process efficiency, representing the combined effect of matrix influence and absolute analyte recovery following extraction, is an important parameter for evaluating and optimizing SPE performance (Cortese et al. 2020). Assuming ion suppression, i.e. a loss of ion intensity of the target analyte, which is the most commonly observed in LC–MS/MS (Panuwet et al. 2016), a PE value below 100% is expected, reflecting both matrix effects and analyte loss during extraction; nevertheless, the use of isotopically labeled internal standards effectively compensated for these variations, ensuring consistent relative recovery and reliable quantification even at low concentration levels, as described in the method validation section.

SPE cartridges did significantly influence PE values for different analytes under the same washing condition (10% methanol in water, v/v, 100%). First, Strata™-X-CW provided the highest PE values for most analytes, followed by Bond Elut Plexa, which provided similar or slightly higher PE compared to Oasis HLB. For example, the PE of IMI-U, a metabolite of IMI, is 38.0 (± 2.5)% with Strata™-X-CW and the values are 17.5 (± 0.6)% and 18.4 (± 0.8)% with Oasis HLB and Bond Elut Plexa, respectively. For CLO-N-DES, a metabolite of CLO, the PE is 36.4 (± 1.5)%, 10.0 (± 0.4)%, and 16.7 (± 0.6)% with Strata™-X-CW, Oasis HLB and Bond Elut Plexa, respectively. This is likely due to Strata™-X-CW’s providing better retention for polar or basic metabolites through combined hydrophobic and weak cation-exchange interactions under neutral conditions (Sadutto et al. 2020), resulting in higher recovery than Oasis HLB or Plexa. Because of the generally higher PE for most analytes, we elected to use the Strata™-X-CW cartridge for this method.

Next, the washing solvent used in the SPE process was optimized. Applying a strong washing solvent during solid-phase extract might minimize matrix effects but lead to analyte loss. Thus, 10–30% methanol in water was compared and the results are shown in Fig. 1. Applying a higher percentage of methanol significantly improved the PE values for most of the analytes, such as ACE, CLO, ACE-N-DES, and IMI-O; however, 30% methanol resulted in the loss of most FLO and CLO-U with PE values ≤ 10% in both cases. Thus, 20% methanol in water was selected as the washing solvent.

Process efficiency reflects the combined influence of extraction recovery and matrix effects and therefore should not be interpreted as extraction recovery alone. During method development, PE was used as an overall indicator to guide optimization of extraction conditions, whereas matrix effects and relative recovery were subsequently evaluated during validation to distinguish ionization effects from extraction-related losses.

#### Matrix effect in synthetic urine vs. pooled urine

Matrix effects (ME) are usually caused by co-eluting components in the matrix that alter the ionization and/or chromatographic response for target analytes (Panuwet et al. 2016). A positive ME value indicates ionization enhancement, and a negative value indicates ionization suppression (Panuwet et al. 2016). Neonicotinoids and their metabolites might experience large matrix effects in urine samples (Honda et al. 2019; Wrobel et al. 2022) because of its complex components such as ions, organic molecules, protein, and water-soluble vitamins (Panuwet et al. 2016). For example, Masato Honda et al. (Honda et al. 2019) reported a large matrix effect from −75 to −90% for THX in extracted urine with four different SPE cartridges.

**Fig. 2:**
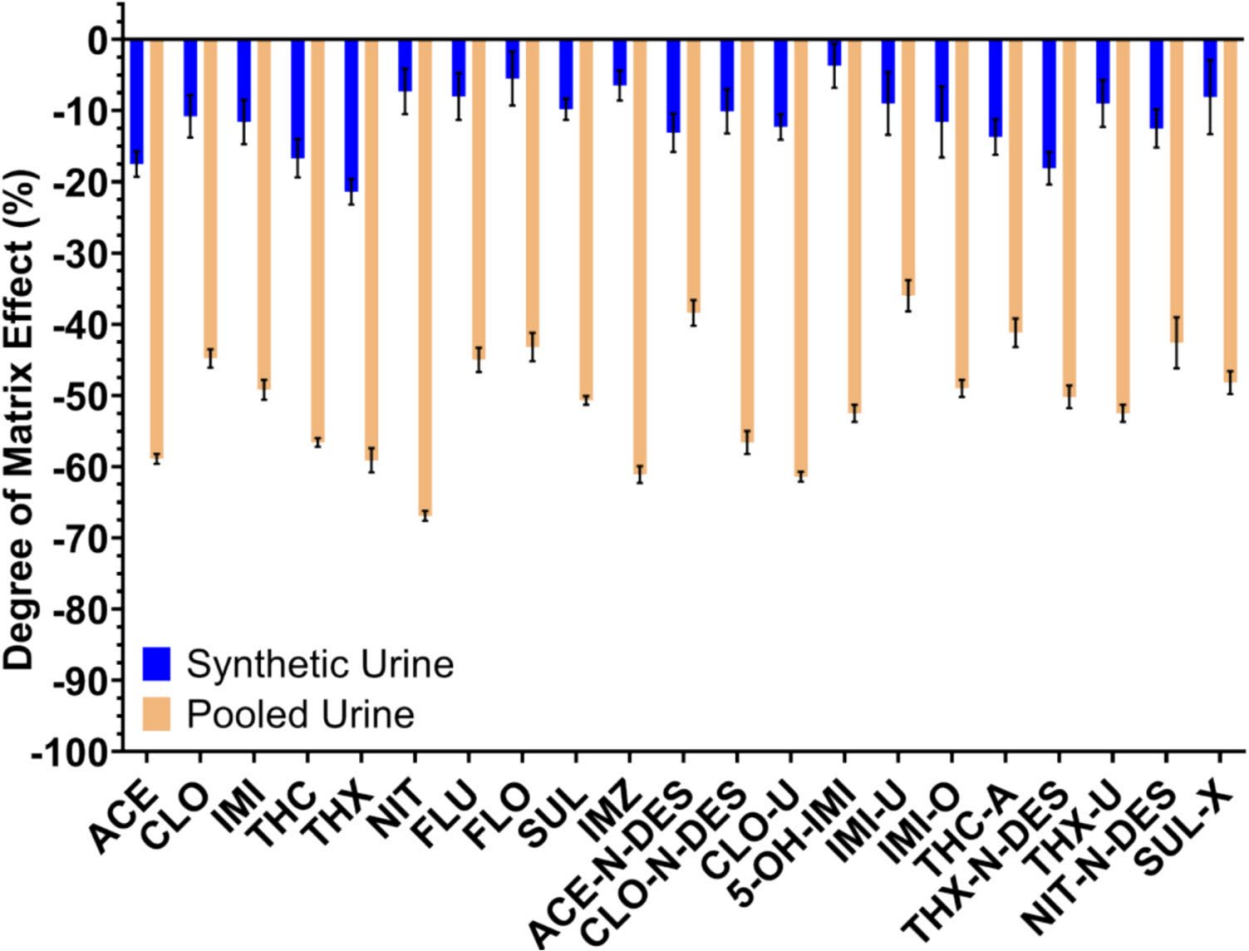
Degree of matrix effect (%) for neonicotinoids and their metabolites in synthetic urine vs. in pooled urine. For all analyses, *n* = 9; error bars represent the standard deviation

In this study, the degree of matrix effects was evaluated for target analytes in both synthetic urine and pooled urine using the optimized extraction method described above. The results are plotted in Fig. 2, and ionization suppression is observed for all analytes in both matrixes. Figure 2 indicates that analytes exhibited minor ion suppression in synthetic urine (−5.5 to −21.4%) whereas substantially stronger ion suppression was observed in pooled urine (−36.0 to −66.9%). Among all analytes, NIT and CLO-U were the most strongly affected by the urine matrix, with a signal suppression of −66.9% and −61.4%, respectively. IMI-U and ACE-N-DES were the least affected, with MEs of −36.0% and −38.4%. The ME comparison in both urine matrixes indicates that pooled urine is a more representative matrix and should be applied for method development and validation, instead of synthetic urine.

Matrix-related ion suppression is expected in pooled urine due to co-eluting endogenous constituents that compete with analytes during electrospray ionization. In the present method, 1 mL urine was diluted with 10 mL water prior to SPE to reduce matrix load while maintaining sufficient analyte mass for reliable quantification. Although a higher dilution factor could further decrease matrix effects, this would proportionally increase the loading volume and processing time and may not be practical for routine use. Similarly, strengthening SPE wash conditions can reduce residual interferences; however, for a wide-coverage method, this must be balanced against the risk of washing off early-eluting analytes and reducing overall analyte coverage. Therefore, the current protocol represents a compromise between minimizing ion suppression and preserving recovery across as many analytes as possible. Further method tuning can be considered on an analyte-by-analyte basis (e.g., modest wash-strength adjustments with recovery monitoring, chromatographic conditions that shift analyte retention away from early-eluting matrix components, or reduced injection volume). In addition, for analytes exhibiting large matrix effects, it is important to use isotopically labeled analogues, when available, of the target analytes as internal standards to correct for these matrix effects (Honda et al. 2019; Panuwet et al. 2016).

To better distinguish extraction-related losses from ionization suppression, apparent recovery was estimated using the relationship RE = PE/(1 + ME), following the matrix-effect evaluation framework described by Matuszewski et al. (Matuszewski et al. 2003). This analysis indicates that reduced PE for some analytes was primarily driven by ion suppression rather than incomplete extraction. For example, ACE exhibited a PE of approximately 38% with a matrix effect of −58%, corresponding to an estimated recovery of about 90%, demonstrating efficient extraction but substantial ion suppression in pooled urine.

#### Method validation

Linearity, endogenous level in matrix blank, LOD, LOQ, precision, and accuracy (relative recovery) were evaluated to assess the method. The results are summarized in Table 2. The calibration curves showed good linearity (*r*^2^ > 0.99) for all analytes over the range from LOD to 25 ng/mL, except for NIT and CLO-U. The linear range for these two analytes was LOD to 10 ng/mL. The LOD and LOQ of this method were determined using pooled urine spiked with a very low concentration (QCL, 0.025 ng/mL) for most of the analytes, except for CLO, IMI, THX, ACE-N-DES, CLO-N-DES, 5-OH-IMI, IMI-O, and THX-U. For these analytes, a significant amount of each was found in the blank pooled urine (Table 2). For example, 0.30 ng/mL of CLO was found in the blank pooled urine so that the concentration of QCL prepared with pooled urine was 0.34 (± 0.038) ng/mL. The calculated LOD and LOQ based on this were 0.11 ng/ml and 0.38 ng/ml, respectively; however, these numbers are artificially high due to the endogenous level. Thus, for analytes with significant endogenous levels in the pooled urine, QCL prepared with synthetic urine was used for LOD and LOQ estimation. For CLO, these numbers are 0.014 ng/ml and 0.045 ng/ml for LOD and LOQ, respectively, which are 10 times lower than those calculated from pooled urine. As summarized in Table 2, LODs are between 0.0050 and 0.14 ng/mL, and LOQs are between 0.017 and 0.46 ng/mL.

**Table 2:** Validation results (ng/mL) for NEOs and their metabolites for synthetic and pooled urine (bold) (*n* = 18)

Analyte	Method detection limits (in synthetic urine)		Practical reporting limits (in pooled urine)		Blank	QCL			QCML			QCMH			QCH		
						Accuracy		Precision	Accuracy		Precision	Accuracy		Precision	Accuracy		Precision
	LOD	LOQ	LOD	LOQ		Ave	SD	RSD%	Ave	SD	RSD%	Ave	SD	RSD%	Ave	SD	RSD%
ACE	0.0050	0.016	0.0070	0.022	ND	*98.1*	*6.5*	*6.6*	*98.0*	*7.0*	*7.1*	*96.8*	*5.8*	*6.0*	*91.3*	*6.5*	*7.2*
						111.6	8.8	8.0	98.3	6.7	6.8	103.0	6.4	6.2	98.9	4.9	5.0
CLO	0.014	0.045	0.11	0.38	0.30	*91.8*	*18.1*	*19.7*	*92.6*	*7.7*	*8.3*	*91.3*	*6.9*	*7.6*	*88.5*	*3.6*	*4.1*
						105.1	11.9	11.3	98.1	14.6	9.9	98.5	9.2	8.8	96.4	8.4	8.6
IMI	0.0060	0.020	0.014	0.047	0.02	*97.3*	*7.9*	*8.3*	*96.5*	*7.2*	*7.5*	*94.4*	*6.3*	*6.6*	*90.0*	*6.4*	*7.1*
						95.1	11.7	11.3	92.9	5.6	5.8	98.8	6.7	6.7	92.9	4.2	4.5
THC	0.0040	0.013	0.021	0.069	ND	*95.7*	*5.2*	*5.4*	*98.1*	*7.0*	*7.1*	*97.4*	*5.5*	*5.7*	*91.8*	*7.4*	*8.0*
						104.3	7.4	22.3	95.4	4.2	5.3	101.2	4.0	3.9	96.8	2.9	3.0
THX	0.0070	0.023	0.032	0.11	0.07	*95.0*	*9.0*	*9.6*	*96.4*	*6.1*	*6.3*	*94.6*	*6.3*	*6.7*	*90.8*	*5.1*	*5.6*
						104.7	12.9	10.4	98.4	11.3	9.4	103.1	6.7	6.2	97.4	4.4	4.5
NIT	0.0070	0.024	0.025	0.083	ND	*104.1*	*9.6*	*9.2*	*95.3*	*4.4*	*4.6*	*94.6*	*3.8*	*4.0*	*92.0*	*6.3*	*6.9*
						79.8	33.2	41.6	107.6	8.5	8.0	100.4	7.2	7.2	97.6	6.2	6.4
FLU	0.0040	0.013	0.032	0.11	ND	*98.7*	*5.1*	*5.2*	*97.6*	*8.2*	*8.4*	*100.2*	*7.4*	*7.4*	*95.0*	*10.1*	*10.6*
						174.1	63.8	21.7	111.1	23.4	19.6	110.7	18.3	16.6	104.1	18.7	18.0
FLO	0.0060	0.020	0.010	0.032	ND	*105.2*	*8.0*	*7.8*	*98.2*	*6.8*	*6.9*	*97.1*	*7.4*	*7.6*	*92.7*	*9.2*	*10.0*
						133.0	13.0	9.8	102.1	6.3	6.2	111.3	7.1	6.4	104.6	5.3	5.1
SUL	0.0060	0.019	0.030	0.10	ND	*106.7*	*7.5*	*7.0*	*98.4*	*5.6*	*5.7*	*96.7*	*6.6*	*6.8*	*93.3*	*8.3*	*8.9*
						158.4	75.8	14.5	118.4	6.4	5.9	124.1	9.4	8.3	115.7	10.2	8.8
IMZ	0.025	0.050	0.14	0.46	ND	*98.1*	*19.9*	*20.4*	*97.4*	*5.4*	*5.5*	*95.9*	*4.7*	*4.9*	*91.2*	*6.0*	*6.5*
						215.6	182.5	84.7	98.5	10.4	10.6	99.1	4.6	4.6	92.5	5.1	5.6
ACE-N-DES	0.0090	0.030	0.033	0.11	0.10	*104.4*	*11.6*	*11.3*	*97.4*	*7.7*	*7.9*	*97.2*	*7.3*	*7.5*	*92.9*	*6.5*	*7.0*
						278.1	234.5	7.5	123.4	19.6	7.0	112.6	6.1	5.9	107.6	5.9	6.0
CLO-N-DES	0.018	0.060	0.056	0.19	0.11	*91.1*	*24.3*	*26.4*	*101.8*	*8.4*	*8.3*	*100.1*	*7.8*	*7.8*	*92.7*	*7.6*	*8.2*
						106.9	15.9	13.0	101.1	11.7	9.7	103.7	7.7	6.8	100.2	5.9	6.5
CLO-U	0.011	0.035	0.053	0.18	ND	*102.3*	*14.0*	*13.9*	*97.5*	*7.4*	*7.6*	*98.7*	*7.4*	*7.5*	*89.6*	*8.1*	*9.0*
						298.7	70.4	23.6	109.5	11.9	10.8	98.7	10.7	10.8	88.8	8.1	9.1
5-OH-IMI	0.0070	0.023	0.081	0.27	0.10	*103.8*	*8.9*	*8.8*	*98.8*	*8.4*	*8.5*	*96.9*	*8.3*	*8.5*	*90.0*	*7.7*	*8.6*
						248.8	225.9	22.1	104.2	10.3	11.1	98.8	5.3	5.8	92.8	5.6	6.5
IMI-U	0.0070	0.024	0.0070	0.024	ND	*86.9*	*9.8*	*11.2*	*86.8*	*8.5*	*9.8*	*88.9*	*5.5*	*6.2*	*87.6*	*9.0*	*10.3*
						34.3	9.9	28.1	81.3	6.9	8.5	96.0	9.1	9.4	92.6	8.4	9.0
IMI-O	0.013	0.024	0.052	0.18	0.10	*100.8*	*9.7*	*9.6*	*100.6*	*5.3*	*5.3*	*99.3*	*6.6*	*6.7*	*96.6*	*8.1*	*8.3*
						108.1	15.8	12.6	101.5	12.8	10.1	101.8	9.1	8.6	99.4	7.8	7.9
THC-A	0.0070	0.023	0.0090	0.030	ND	*93.7*	*9.1*	*9.8*	*97.5*	*5.8*	*6.0*	*97.2*	*5.8*	*6.0*	*92.1*	*7.6*	*8.3*
						112.1	12.0	10.7	95.2	5.3	5.6	99.2	4.6	4.7	97.8	5.5	5.6
THX-N-DES	0.0080	0.025	0.031	0.10	ND	*97.4*	*10.2*	*10.3*	*97.0*	*7.1*	*7.4*	*95.0*	*4.6*	*4.8*	*90.3*	*5.2*	*5.8*
						108.8	40.9	37.6	98.2	8.4	8.6	103.1	7.1	6.9	95.4	6.8	7.2
THX-U	0.0050	0.017	0.037	0.12	0.13	*85.4*	*7.0*	*8.1*	*82.1*	*7.1*	*8.7*	*85.1*	*7.1*	*8.3*	*81.6*	*7.9*	*9.6*
						101.9	14.4	8.3	90.5	8.8	8.9	86.7	4.5	5.4	83.1	5.7	6.8
NIT-N-DES	0.011	0.035	0.021	0.069	ND	*61.1*	*13.9*	*23.0*	*61.5*	*8.6*	*13.9*	*64.6*	*9.9*	*15.3*	*63.2*	*10.8*	*17.1*
						105.4	27.7	26.2	81.1	10.6	13.1	87.9	11.5	13.1	91.3	10.9	12.0
SUL-X	0.010	0.034	0.026	0.087	ND	*91.1*	*13.7*	*15.0*	*91.6*	*7.9*	*8.6*	*92.0*	*7.2*	*7.8*	*83.9*	*6.1*	*7.3*
						197.5	83.3	14.2	115.7	16.1	13.9	109.1	16.3	14.9	104.4	9.3	8.9

In synthetic urine, the accuracy of QCML (0.25 ng/mL) ranged from 92.6 to 98.4% for neonicotinoid (NEO) parent compounds and 82.1–101.8% for their metabolites, with precision values below 10% for all analytes. The accuracy ranged from 91.3 to 100.2% and 85.1 to 100.1% for parents and metabolites, respectively, with precision less than 8.5% for the medium-high concentration level (QCMH, 1.5 ng/mL). For QCH (4.5 ng/mL), accuracy was 88.5–95.0% and 81.6–96.6% for parents and metabolites, respectively, and precision remained below 10.6%. Recoveries in synthetic urine for the lowest level tested (QCL, 0.025 ng/mL) were also within the acceptable range (70–130%), ranging from 85.4 to 106.7% with precision below 20.4%, except for CLO-N-DES, which showed a precision of 26.4%.

In pooled urine, aided by isotopically labeled analogues, both accuracy and precision for QCML, QCMH, and QCH were comparable to those obtained in synthetic urine and remained within the acceptance criteria (accuracy 70–130%; precision < 20%) for all analytes. For example, at QCML (0.25 ng/mL), accuracy ranged from 92.9 to 118.4% for NEO parents and 81.3–123.4% for metabolites, with precision below 20%. At the lowest level (QCL, 0.025 ng/mL), acceptable accuracy and precision were still achieved for eight analytes—ACE, CLO, IMI, THX, THX-U, THC-A, CLO-N-DES, and IMI-O—whereas the remaining compounds were less reliably quantified due to matrix effects and/or detectable endogenous levels in the blank pooled urine.

It should be noted that LOD and LOQ values can differ substantially depending on the calculation approach used (Anai et al. 2021; Baker et al. 2019; Honda et al. 2019; Zhang & Lu 2022); therefore, these limits are best verified experimentally through assessments of accuracy and precision. For instance, an LOQ of 0.022 ng/mL was estimated for ACE and subsequently confirmed by analyzing the QCL prepared at 0.025 ng/mL, which yielded acceptable accuracy and precision values of 111.6 (± 8.8)% and 8.0%, respectively.

As described in the “Quality controls” section, QC materials prepared from pooled urine were analyzed along with unknown samples. The results were monitored for two months (Table S6) and the mean concentration M_c_ and relative standard deviation RSD% were used to evaluate the inter-day imprecision. All RSD results were below 20%, which indicates that the method is reliable.

The method parameters established in this study are comparable to, or exceed, those reported in other analytical approaches developed for the detection of neonicotinoids (NEOs) in urine. In one previous study (Baker et al. 2019), six common NEO biomarkers were measured using online solid-phase extraction, with reporting LODs ranging from 0.010 to 0.10 ng/mL, while the QCL was prepared at 1.6–4 ng/mL. Another study (Wrobel et al. 2023) developed an analytical method for more analytes, which includes 7 NEOs and NEO-like compounds and 9 metabolites, and that study reported LODs ranging from 0.020 to 0.64 ng/mL. The same study reported their LOD as 0.64 ng/mL for IMI-O compared to 0.0070 ng/mL for IMI-O in our study. This might be due to ESI negative ion mode, instead of positive mode was applied for IMI-O in our study, which led to a much higher sensitivity.

*LOD*, limits of detection; *LOQ*, limit of quantification; *Blank*, averaged endogenous level in the pooled urine (blank, ng/mL); *QCL (QC low)*, 0.025 ng/mL; *QCML (QC medium low)*, 0.25 ng/mL; *QCMH (QC medium high)*, 1.5 ng/mL; and *QCH (QC high)*, 4.5 ng/mL. For analytes with measurable endogenous background in pooled urine, LOD and LOQ values determined in synthetic urine were used to represent method detection capability. Pooled urine values reflect reporting limits under real sample conditions. LOD and LOQ values are reported using two significant figures.

#### Biomonitoring results for pregnant women

**Fig. 3:**
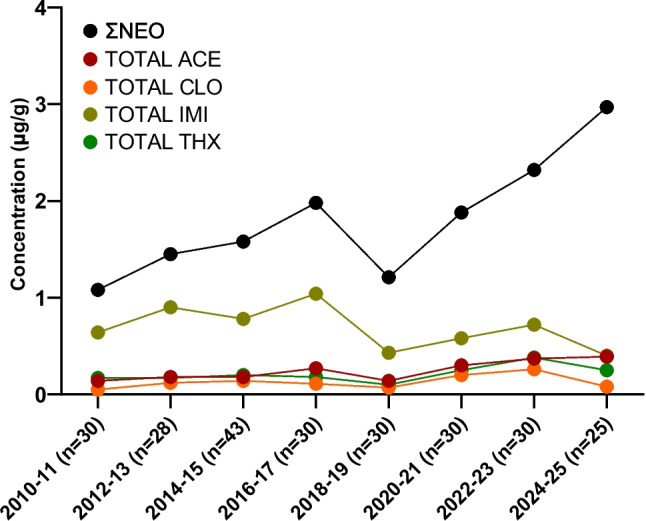
Median concentrations (μg/g) of neonicotinoids in pregnant women in Iowa from 2010 to 2025

NEOs were detected in urine of nearly every pregnant woman tested (*n* = 246), with only two women showing no detectable exposure. A statistically significant increasing trend in median ΣNEO concentrations was observed from 2010 to 2025 when assessed by 2-year intervals (Kruskal-Wallis *p* = 0.044; Spearman *r*_s_ = 0.165, *p* = 0.010) (Fig. 3).

This increase was not driven by higher concentrations of the major parent compounds like IMI and CLO, which remained relatively stable across the study period. Kruskal-Wallis tests showed no significant difference in median concentrations by year for IMI (*p* = 0.533) and CLO (*p* = 0.315). Spearman also indicated no correlation between concentrations and time (*p* > 0.05). Instead, this temporal rise in ΣNEO was primarily attributable to significantly increasing contributions of THX, ACE-N-DES, FLU, and SUL. Each of these analytes showed significant differences in median concentrations over time (THX: Kruskal-Wallis *p* = 0.002; Spearman *r*_s_ = 0.157, *p* = 0.014; ACE-N-DES**:** Kruskal-Wallis *p* = 0.037; Spearman *r*_s_ = 0.185, *p* = 0.004; FLU: Kruskal-Wallis *p* = 0.026; Spearman *r*_s_ = 0.191, *p* = 0.003; and SUL: Kruskal-Wallis *p* = < 0.001; Spearman *r*_s_ = 0.195, *p* = 0.002). This trend was largely due to increases in these specific compounds and the number of chemicals detected in samples, rather than higher concentrations of the main NEOs CLO and IMI. These trends were also observed when assessing the aggregate metrics with ƩTHX and ƩACE showing significant increasing trends compared to ƩIMI and ƩCLO, which remained stable.

Median ƩNEO concentrations in 2010–2011 were 1.1 μg/g compared to 3.0 μg/g in 2024–2025. There was a greater variability in the average concentrations at 3.1 μg/g (max = 26.3) in 2010–2011 compared to 3.7 μg/g (max = 12.4). Eighteen different NEOs and metabolites were detected across all the pregnant women tested. Three analytes (THC, THC-A, and NIT-N-DES) were not detected in any sample. The median number of NEOs and metabolites detected per urine sample was 5 and the max number of analytes per sample was 12. The median number of detects per sample increased significantly (*p* = 0.017) over time, with the median number of NEOs detected per sample rising from 4.5 in 2010–2011 to 6 in 2024–2025.

Table 3 summarizes the urinary concentrations of NEOs and their metabolites among 246 pregnant women in Iowa from 2010 to 2025. For each compound, the table reports detection frequency (DF%), mean concentration, standard deviation, 95% confidence interval, median, and maximum values, providing a comprehensive overview of exposure levels in this population. As shown in Table 3, the maximum detectable total concentrations of NEOs and metabolites (ƩNEO**)** ranged from non-detected to 102.0 μg/g with an average of 4.6 μg/g. Two metabolites, ACE-N-DES (max: 41.9 μg/g) and IMI-O (max: 30.6 μg/g), were detected in more than 85% of samples. CLO, CLO-N-DES, IMI, 5-OH-IMI, and THX were all detected in over 40% of maternal urine samples.

**Table 3:** Concentration (μg/g) of NEOs and metabolites in urine samples from pregnant women in Iowa from 2010 to 2025 (*n* = 246)

Analyte	Total DF% (number)	Mean	Std Dev	Lower 95%	Upper 95%	Median	75%	90%	Max	*p*-value*
ACE	13.0 (32)	0.01	0.02	<	0.01	<	<	0.02	0.14	0.354
CLO	51.2 (126)	0.38	1.32	0.21	0.55	0.07	0.3	0.74	17.04	0.315
IMI	43.5 (107)	0.13	0.53	0.06	0.2	<	0.08	0.27	6.93	0.533
THX	48.0 (118)	0.19	0.69	0.1	0.27	0.01	0.13	0.35	8.82	0.002
NIT	6.1 (15)	0.01	0.04	0.01	0.02	<	<	0.01	0.38	0.123
FLU	13.4 (33)	0.11	0.65	0.02	0.19	<	0.01	0.15	8.21	0.026
FLO	9.8 (24)	0.2	1.28	0.04	0.36	<	<	0.05	16.99	0.565
SUL	19.9 (49)	0.1	0.61	0.02	0.17	<	0.01	0.18	9.07	0
IMZ	0.8 (2)	0.03	0.14	0.01	0.05	0.01	0.02	0.03	2.12	0.827
ACE-N-DES	86.6 (213)	1.17	3.97	0.68	1.67	0.21	0.67	2.31	41.94	0.037
5-OH-IMI	68.3 (168)	0.85	2.54	0.53	1.17	0.2	0.6	1.53	25.68	0.239
THX-N-DES	2.4 (6)	0.03	0.13	0.01	0.04	<	0.01	0.04	1.86	0.741
CLO-U	2.0 (5)	0.01	0.05	<	0.02	<	0.01	0.01	0.8	0.634
THX-U	15.5 (38)	0.01	0.04	<	0.01	<	0.01	0.01	0.47	0.764
IMI-U	9.3 (23)	0.08	1.05	<	0.21	<	<	0.01	16.42	0.161
CLO-N-DES	48.0 (118)	0.14	0.38	0.09	0.19	0.02	0.11	0.33	4.1	0.797
SUL-X	12.6 (31)	0.04	0.13	0.02	0.05	<	0.01	0.06	1.25	0.09
IMI-O	93.1 (229)	1.11	2.94	0.74	1.48	0.35	0.9	2.43	30.6	0.216
ƩNEO	99.2 (244)	4.58	10.25	3.29	5.87	1.61	4.1	8.5	102	0.044
Detects/Sample		5.44	2.1	5.17	5.7	5.5	7	8	12	0.017

THC, THC-A, and NIT-N-DES not detected

*Kruskal-Wallis comparison by 2-year increments<, below limit of detection

*n.d.*, not detected

*DF*, detection frequency

ACE-N-DES is commonly reported as the most frequently detected metabolite in every paper reviewed (Baker et al. 2019; Ospina et al. 2019; Pan et al. 2023). Here, ACE-N-DES was detected in 86.6% of samples with an average concentration of 1.17 μg/g. Other studies of pregnant populations have found ACE-N-DES in high detection frequencies in maternal serum samples, umbilical cord samples, and breast milk samples (Chen et al. 2020; Huang et al. 2024; Zhang et al. 2022). Median concentrations of ACE-N-DES showed a statistically significant increase with time (Kruskal-Wallis *p* = 0.037) from 0.12 μg/g in 2010–2011 to 0.37 μg/g in 2022–2023 and 0.39 μg/g in 2024–2025.

In this work, IMI-O, which has been rarely measured in published studies, was the most frequently detected analyte. IMI-O was detected in 93.1% of samples with an average concentration of 1.2 μg/g. IMI-O concentrations were not significantly correlated with time (Spearman *r*_s_ = −0.073, *p* = 0.257), suggesting that concentrations did not significantly change across the 15-year time period. Median concentrations were also not different by 2-year periods (Kruskal-Wallis *p* = 0.216). Other studies have measured IMI-O in pregnant populations, and it was associated with oxidative stress biomarkers (Wang et al. 2024; Zhang et al. 2022). Of note, structural alteration of NEOs is often associated with decreased toxicity to vertebrates; however, IMI-O is significantly more insecticidal than the IMI (Dai et al. 2006). Median concentrations of IMI (*p* = 0.533) and 5-OH-IMI (*p* = 0.239), another IMI metabolite, did not vary significantly over time. The ƩIMI-related concentrations (IMI + IMI-O + 5-OH-IMI + IMI-U) also were not significantly different (Kruskal-Wallis *p* = 0.453; Spearman *r*_s_ = −0.043, *p* = 0.507) over time. 

Median urinary concentrations differed significantly over time for FLU (*p* = 0.026) and SUL (*p* = < 0.001). FLU was detected in nine (5.6%) samples prior to 2020 but was found in 24 (28%) samples from 2020 to 2025. FLU was detected in forty percent of the samples tested since 2023. Meanwhile the concentrations of SUL and its metabolite SUL-X were also higher in later samples. Average concentrations for SUL and SUL-X were 0.01 μg/g (max = 0.20) and 0.02 μg/g (max = 0.33) in 2010–2011 compared to 0.24 μg/g (max = 1.71) and 0.05 μg/g (max = 0.60) in 2024–2025, respectively. Detection frequencies were 10% and 3% in 2010–2011 versus 48% and 20% for SUL and SUL-X, respectively.

This study found that NEO and metabolite concentrations were generally positively correlated (Spearman) with one another, potentially suggesting common source or sources of exposure (Table S7). Among all possible analyte pairs (*N* = 246), 94.8% showed statistically significant positive correlations (*p* < 0.05), indicating pervasive co-occurrence likely reflecting shared dietary or environmental exposure pathways. In particular, the parent compounds ACE, CLO, THX, and IMI showed strong positive correlations across analytes and detection counts (*p* < 0.0001), reflecting consistent co-occurrence patterns and potentially common environmental pathways.

CLO showed its strongest parent-compound association with THX (*r*_s_ = 0.388, *p* < 0.0001) and was also strongly correlated with total detection counts (*r*_s_ = 0.543, *p* < 0.0001), suggesting that CLO presence may reflect broader multi-compound exposure. IMI correlated moderately but significantly with ACE (*r*_s_ = 0.454, *p* < 0.0001), CLO (*r*_s_ = 0.247, *p* < 0.0001), and THX (*r*_s_ = 0.252, *p* < 0.0001), consistent with co-occurrence across multiple neonicotinoid classes.

Metabolites provided additional exposure information beyond simply tracking their parent compounds. 5-OH-IMI and IMI-O were strongly intercorrelated (*r*_s_ = 0.698, *p* < 0.0001), and each correlated significantly with parent IMI (5-OH-IMI: *r*_s_ = 0.616, *p* < 0.0001; IMI-O: *r*_s_ = 0.526, *p* < 0.0001). ACE-N-DES, the n-desmethyl metabolite of acetamiprid, correlated significantly with parent ACE (*r*_s_ = 0.243, *p* < 0.0001) and showed broad associations across the analyte matrix. Notably, IMI-O showed the strongest correlation with total neonicotinoid burden across all analytes (*r*_s_ = 0.735 with ΣNEO, *p* < 0.0001), followed by 5-OH-IMI (*r*_s_ = 0.698, *p* < 0.0001) and ACE-N-DES (*r*_s_ = 0.300, *p* < 0.0001). These metabolites outperformed all parent compounds as predictors of cumulative exposure, suggesting that metabolite monitoring, particularly of imidacloprid metabolites, may provide a more sensitive measure of overall neonicotinoid exposure in pregnant women than measurement of parent compounds alone.

One limitation of the analysis of samples from pregnant women was that individual-level metadata were limited by IRB requirements, as the project was designed for analytical method development rather than epidemiologic inference, precluding evaluation of sociodemographic or lifestyle factors. Future work will incorporate more detailed participant data and larger sample sizes to better characterize exposure determinants and health risks.

#### Biomonitoring results for male farmers

In our prior study, we reported that NEOs were ubiquitous in urine samples from farmers in eastern Iowa (Thompson et al. 2023). Urine from farmers showed widespread exposure to a variety of NEOs, with all samples collected containing at least six detectable analytes. That study reported the median number of NEOs detected per urine sample was 10, with a range of 6–13 detections across all samples.

In this study, first morning void samples were retested for nine new analytes, including FLU and eight metabolites (CLO-N-DES, CLO-U, 5-OH-IMI, IMI-U, IMI-O, NIT-N-DES, SUL-X, and THX-U) not measured previously. These samples were retested primarily to assess whether IMI-O exposure was as common among this farming cohort as pregnant women*.* The researchers wanted to determine whether IMI-O was unique to pregnant women or if it was also present among a broader set of Iowans.

Using previously reported data plus the results from these new analytes, we found that median ƩNEO differed significantly when comparing farmers to pregnant women. As shown in Table 4, the median ƩNEO concentration was 3.9 μg/g for farmers compared to 1.6 μg/g for pregnant women (Wilcoxon Rank Sum *p* = < 0.0001). Farmers also had a significantly higher mean number of analyte detections per sample (13.96 vs. 5.44). The detection profiles differed markedly between cohorts: THC-A, CLO, THX, NIT, and IMI-O were each detected in 91–100% of farmer samples, while several analytes including ACE, NIT, FLU, THC, and THC-A were rarely or never detected in pregnant women. IMZ was detected in 98% of farmer samples at substantially higher concentrations than in pregnant women (median 1.06 vs. 0.01 μg/g, *p* < 0.0001).

**Table 4:** Concentration (μg/g) of NEOs and metabolites in urine samples from farmers in Iowa from 2018 to 2019 (*n* = 47)

Analyte	DF (%)	Mean	Std Dev	Lower 95%	Upper 95%	Median	75%	0.90%	Max	*p*-value*
ACE	9	0.02	0.02	0.01	0.02	0.01	0.01	0.04	0.11	< 0.0001
CLO	100	0.39	0.55	0.23	0.55	0.2	0.39	0.74	3.26	< 0.0001
IMI	53	0.59	1.32	0.21	0.98	0.08	0.54	2.12	7.42	< 0.0001
THC	19	0.02	0.05	0.00	0.03	0.01	0.01	0.03	0.36	< 0.0001
THX	100	0.41	0.49	0.27	0.56	0.3	0.41	0.73	3.35	< 0.0001
NIT	100	0.2	0.16	0.15	0.24	0.16	0.22	0.4	0.72	< 0.0001
FLU	< 1	0.02	0.13	<	0.06	<	<	<	0.92	0.03
FLO	36	0.03	0.05	0.02	0.05	0.01	0.03	0.09	0.28	< 0.0001
SUL	23	0.03	0.10	0.00	0.06	0.01	0.02	0.04	0.7	< 0.0001
IMZ	98	1.79	2.27	1.12	2.46	1.06	1.76	5.23	10.3	< 0.0001
ACE-N-DES	89	0.37	0.76	0.15	0.60	0.07	0.21	1.19	3.43	0.006
5-OH-IMI	51	0.21	0.40	0.10	0.33	0.03	0.27	0.63	1.69	0.016
THX-N-DES	91	0.17	0.21	0.11	0.24	0.1	0.23	0.47	1.25	< 0.0001
CLO-U	n.d	<	<	<	<	<	<	<	<	0.327
THX-U	11	0.01	0.03	0.00	0.02	<	<	0.02	0.17	0.433
IMI-U	n.d	<	<	<	<	<	<	<	<	0.03
THC-A	100	0.3	0.26	0.23	0.38	0.22	0.39	0.51	1.51	< 0.0001
CLO-N-DES	32	0.2	0.79	−0.04	0.43	<	0.09	0.44	5.34	0.252
SUL-X	n.d	<	<	<	<	<	<	<	<	0.01
IMI-O	100	0.59	0.90	0.32	0.85	0.29	0.67	1.41	5.44	0.532
ƩNEO*	100	5.36	4.57	4.02	6.70	3.93	5.98	12.74	25.64	< 0.0001
Detects/Sample		13.96	0.78	13.73	14.19	14	14	15	16	< 0.0001

*Wilcoxon Rank Sum comparison between farmers and pregnant women

**6-CN, DIN were not included in the ƩNEO or Detects/Sample calculated for comparison purposes. Neither analyte was included in the method described in this publication

<, below limit of detection

*n.d.*, not detected

*DF*, detection frequency

CLO-U, IMI-U, SUL_X, and NIT-N-DES were not detected in the samples from farmers

Despite farmers having a higher overall neonicotinoid burden, three analytes—ACE-N-DES, CLO-N-DES, and 5-OH-IMI—showed higher concentrations in pregnant women. These analytes were detected in 32%, 89%, and 51% of farmers’ samples compared to 87%, 48%, and 68% of pregnant women’s samples, respectively. Median concentrations of ACE-N-DES and 5-OH-IMI were significantly elevated in pregnant women: 0.21 μg/g vs. 0.07 μg/g for ACE-N-DES (*p* = 0.006) and 0.20 μg/g vs. 0.03 μg/g for 5-OH-IMI (*p* = 0.016). CLO-N-DES also showed higher median concentrations in pregnant women (0.02 μg/g vs. < LOD in farmers), though this difference was not statistically significant (*p* = 0.252). Concentrations at the 75th and 90th percentiles were nearly double in pregnant women for ACE-N-DES and 5-OH-IMI. Maximum concentrations were also higher in pregnant women across analytes, a pattern largely driven by individual outliers.

IMI-O was found in 100% of farmers’ samples compared to 93% of pregnant women’s samples. Median concentrations, however, showed no statistical difference between groups (0.29 μg/g in farmers vs. 0.35 μg/g in pregnant women, *p* = 0.532). This finding is notable given the substantially different exposure contexts of the two cohorts and suggests that IMI-O production may be a common feature of imidacloprid metabolism regardless of exposure route or intensity. This implies that IMI-O may be indicative of a common exposure to IMI among Iowans. More research is needed to assess whether similar exposures may be prevalent across the USA.

#### Comparison between male farmers and pregnant women

In the 2023 study, it was hypothesized that farmers would represent a potential worst-case scenario for exposure due to their history of NEO use and consumption of drinking water from sources vulnerable to contamination (Thompson et al. 2023). However, these findings suggest that the level of exposure may be comparable across different vulnerable groups with 99% of pregnant women and 100% of farmers having a detectable concentration of at least one NEO in their sample. In some instances, such as with ACE-N-DES and 5-OH-IMI, concentrations were significantly higher among pregnant women than farmers, while there was no difference in concentrations of IMI-O between the two groups.

NEOs and metabolite concentrations showed different correlation patterns between the two cohorts, suggesting different exposure sources or pathways (Tables S7–8**).** In pregnant women, 94.8% of analyte pairs were significantly correlated (*p* < 0.05), indicating widespread co-occurrence likely from shared dietary or environmental sources. In farmers, only 44.6% of pairs reached significance, suggesting that occupational exposures could be more variable, with individual compounds exposure taken place independently.

Among parent compounds, pregnant women showed strong positive correlations across most analytes (*p* < 0.0001). ACE formed particularly strong associations with NIT (*r*_s_ = 0.733), FLU (*r*_s_ = 0.725), and FLO (*r*_s_ = 0.749), indicating these compounds frequently co-occur from common exposure sources. In farmers, correlations were more selective. CLO and THX showed a strong association (*r*_s_ = 0.582, *p* < 0.0001), consistent with co-application in occupational settings. However, IMI showed weak or non-significant correlations with other parent compounds in farmers, including ACE (*r*_s_ = 0.107, *p* = 0.472), FLU (*r*_s_ = −0.091, *p* = 0.542), and CLO (*r*_s_ = −0.025, *p* = 0.865), contrasting with the moderate but significant correlations observed in pregnant women.

In pregnant women, 5-OH-IMI and IMI-O were strongly intercorrelated (*r*_s_ = 0.698, *p* < 0.0001) and each correlated significantly with IMI (5-OH-IMI: *r*_s_ = 0.616, *p* < 0.0001; IMI-O: *r*_s_ = 0.526, *p* < 0.0001). In farmers, 5-OH-IMI showed no significant correlation with IMI (*r*_s_ = −0.082, *p* = 0.582) but showed strong association with IMI-O (*r*_s_ = 0.591, *p* < 0.0001) and detection counts (*r*_s_ = 0.730, *p* < 0.0001).

IMI-O showed the strongest correlation with total neonicotinoid burden in pregnant women (*r*_s_ = 0.735 with ΣNEO, *p* < 0.0001), followed by 5-OH-IMI (*r*_s_ = 0.698, *p* < 0.0001) and ACE-N-DES (*r*_s_ = 0.300, *p* < 0.0001). In farmers, the IMI-O correlation with ΣNEO was weaker (*r* = 0.449, *p* = 0.002). These metabolites outperformed all parent compounds as predictors of cumulative exposure in pregnant women, suggesting that metabolite monitoring, particularly of imidacloprid metabolites, may provide more sensitive measures of overall neonicotinoid exposure than parent compounds alone.

Several factors should be considered when interpreting between-cohort differences. Parent neonicotinoids were measured using different laboratory methods across cohorts, while the nine metabolites were analyzed using a common method, providing a more consistent basis for metabolite comparisons. Additionally, pregnant women’s samples spanned 2010–2025, while farmer samples were collected during a narrow window (December 2018–March 2019). This temporal difference may account for some observed variations, as the extended collection period encompasses considerable changes in neonicotinoid use patterns.

#### Analytical stability and temporal patterns in neonicotinoid detection

Urine samples were processed across 11 LC/MS analytical batches of varying size (3–46 samples per batch) between September 2024 and May 2025. Batch assignment was not randomized. Samples were processed as received box-by-box as provided by PFTB. Each box contained specimens from multiple collection years. QC and matrix blanks were run with each batch to track method performance during sample runs.

Statistical analysis of analyte concentrations by batch and collection period revealed distinct patterns depending on the compound (Tables S4–S5). Metabolites, particularly IMI-O and other imidacloprid metabolites, showed remarkable stability across analytical batches and time periods. IMI-O showed no significant batch effects (Kruskal-Wallis *p* = 0.157) and negligible correlations with both batch (*r*_s_ = 0.046, *p* = 0.474) and collection year (*r*_s_ = −0.073, *p* = 0.257). Similarly, 5-OH-IMI showed no significant batch variation (*p* = 0.088), and ACE-N-DES, the second most frequently detected analyte, showed no significant association with batch processing date (*p* = 0.070), despite spanning 15 years of sample collection. This stability was observed despite substantial variation in batch sample sizes and collection periods, suggesting these metabolite concentrations reflect genuine biological exposure rather than analytical artifacts.

In contrast, several parent compounds with higher detection frequencies, including IMI and THX—exhibited significant median concentrations by batch (*p* < 0.05), but temporal batch trends were not correlated with their concentrations (IMI: *r*_s_ = −0.021, *p* = 0.744; THX: *r*_s_ = −0.117, *p* = 0.066). THX and ƩTHX concentrations did have a positive correlation with the date samples collected across annual, 2-year, and 5-year periods (*p* < 0.05). CLO, on the other hand, was negatively correlated with batch date (*r*_s_ = −0.178, *p* = 0.005), but were not different in median concentrations were observed by batch (*p* = 0.291). Concentrations of CLO, ƩCLO, IMI, and ƩIMI did not differ by date of sample collection for any of the time periods analyzed. Given that these are parent compounds, temporal differences likely reflect changes in real-world exposure patterns over time, such as shifting agricultural practices or regulatory changes, rather than storage- or processing-related artifacts.

Analytes with low detection frequencies (SUL, FLU, NIT, SUL-X; detection rates 6–19%) showed greater apparent variability, consistent with limited data available for statistical comparison. However, formal testing confirmed that much of this apparent variation reflected random differences rather than systematic batch-related effects.

The unexpectedly high detection frequency of IMI-O also prompted a series of targeted evaluations to determine whether these concentrations reflected true exposure or could be explained by analytical artifacts, in-source conversion, or degradation during storage. Batch-to-batch comparisons demonstrated minimal variability, indicating that instrument performance was unlikely to account for the frequent detection of IMI-O. The statistical analysis provided strong evidence against storage or processing artifacts: no temporal trends were observed across the 2010–2025 period (Kruskal-Wallis *p* = 0.216), with Spearman correlation confirming negligible associations for both batch (*r*_s_ = 0.046, *p* = 0.474) and year (*r*_s_ = − 0.049, *p* = 0.444). All biospecimens were stored continuously at −80 °C prior to extraction. We were unable to find published evidence suggesting instability of NEOs or their metabolites at this temperature. Although chemical transformation can occur at higher temperatures due to metabolic or microbial activity (Thompson et al. 2020), such reactions are not expected under deep-freeze conditions.

In 2025, 15 newly collected samples were analyzed and compared with older specimens, further confirming that IMI-O likely did not form during long-term storage or sample processing. The statistical stability of IMI-O across 11 analytical batches, 15 + years of collection time, and varying analytical conditions strengthens confidence that detected concentrations represent genuine exposure Despite this evidence, further research on storage stability is needed, as the absence of extensive long-term data means that minor storage-related effects cannot be entirely ruled out.

## Conclusions

This study successfully developed and validated a comprehensive LC–MS/MS method for quantifying 21 NEOs and their metabolites. The method showed good analytical performance, with LOD ranging from 0.0050 to 0.14 ng/mL and LOQ from 0.017 to 0.46 ng/mL. Across quality control levels, the method demonstrated reliable quantitative performance, with accuracies within 70–130% and precision generally below 20% in both synthetic and pooled urine, while a few analytes at the lowest level were affected by matrix effects or endogenous background. The method is therefore well suited for biomonitoring applications, particularly because it enables simultaneous measurement of multiple metabolites that are often not included in previous studies.

Analysis of urine samples from pregnant women and farmers in Iowa indicated near-ubiquitous exposure to multiple neonicotinoids (NEOs) and their metabolites across both groups. The findings underscore the importance of comprehensive biomonitoring methods to accurately assess human exposure to NEOs, particularly in regions with intensive agricultural practices. Notably, metabolites such as IMI-O and 5-OH-IMI served as stronger predictors of total neonicotinoid burden than parent compounds alone. Additionally, some metabolites like ACE-N-DES and 5-OH-IMI showed higher concentrations in pregnant women even though farmers had significantly higher total neonicotinoid exposure.

Further research is needed to understand the health implications of such exposure, especially among vulnerable groups like pregnant women. Overall, this study highlights the need for ongoing monitoring of NEOs to mitigate potential health risks associated with their widespread use. The validated method provides a valuable tool for future studies aimed at understanding the extent and impact of NEO exposure on human health.

## Supplementary Information

Below is the link to the electronic supplementary material.

ESM 1 (DOCX 1.22 MB)

## Data Availability

The datasets generated and analyzed during the current study are not publicly available due to participant privacy protection but are available from the corresponding author on reasonable request.
