# Supplementary material for: Development and validation of matrix-validated LC–MS/MS method for simultaneous quantification of 21 neonicotinoids and their metabolites in human urine
Source: Environ Sci Pollut Res Int. 2026 May 1;33(16):7576–93. doi: 10.1007/s11356-026-37779-9 (PMC13190543; doi:10.1007/s11356-026-37779-9)
Supplement: Supplementary file 1 — (DOCX 1.22 MB) [file 11356_2026_37779_MOESM1_ESM.docx]

**Supporting Information**

**Development and Validation of Matrix‑Validated LC‑MS/MS Method for Simultaneous Quantification of 21 Neonicotinoids and their Metabolites in Human Urine**

Wenjing Xi^a^*, Sarah Onysio^a^, Mary C. Rhodes^b^, Alexis J. Faudel^c^, Mark Santillan^c^, Donna Santillan^c^, David M. Cwiertny^ade^, and Darrin A. Thompson^ab^

^a^ University of Iowa, Center for Health Effects of Environmental Contamination, Iowa City, IA, USA

^b^ University of Iowa, College of Public Health, Iowa City, IA, USA

^c^ University of Iowa, Department of Obstetrics and Gynecology, Iowa City, IA USA

^d^ University of Iowa, Department of Civil & Environmental Engineering, Iowa City, IA, USA

^e^ University of Iowa, Department of Chemistry, Iowa City, IA, USA

**Table S1.** Acronym, manufacturing, CAS number, molecular weight (MW), and the assigned isotope labelled internal standard for each target analyte.

| **Compound** | **Acronym** | **Manufacturing** | **CAS Number** | **MW** | **Internal Standard** |
| --- | --- | --- | --- | --- | --- |
| Acetamiprid | ACE | Chem Service | 135410-20-7 | 208.65 | ACE-d3 |
| *Acetamiprid-n-desmethyl* | ACE-N-DES | HPC | 190604-92-3 | 208.65 | 3-^2^H,^13^C, 2-^15^N-ACE-N-DES |
| Clothianidin | CLO | Chem Service | 210880-92-5 | 249.68 | CLO-d3 |
| *Clothianidin-n-desmethyl* | CLO-N-DES | HPC | 135018-15-4 | 235.65 | CLO-N-DES (Guanidine-^13^C, Guanidine-1,3-^15^N_2_) |
| *Clothianidin urea (thiamethoxam metabolite CGA 353968)* | CLO-U | HPC | 634192-72-6 | 205.67 | THX-d3 |
| Imidacloprid | IMI | Chem Service | 138261-41-3 | 255.66 | IMI-d4 |
| *5-hydroxy-imidacloprid* | 5-OH-IMI | CIL^*^ | 155802-61-2 | 271.66 | 5-OH-IMI (2-^13^C, 3-^15^N, 2-amino-^15^N) |
| *Imidacloprid urea* | IMI-U | HPC | 120868-66-8 | 211.65 | 3-^2^H,^13^C, 2-^15^N-ACE-N-DES |
| *Imidacloprid olefin* | IMI-O | HPC | 115086-54-9 | 253.65 | IMI-O (Imidazol-1-^15^N, 2-^13^C, 2-amino ^15^N) |
| Thiacloprid | THC | Chem Service | 111988-49-9 | 252.72 | THC-d4 |
| *Thiacloprid amide* | THC-A | Sigma Aldrich | 676228-91-4 | 270.74 | THC-A (pyridylmethyl-^13^C_6_) |
| Thiamethoxam | THX | Chem Service | 153719-23-4 | 291.71 | THX-d3 |
| *Thiamethoxam-n-desmethyl* | THX-N-DES | Chem Service | 171103-04-1 | 277.69 | IMZ-d4 |
| *Thiamethoxam urea (thiamethoxam metabolite CGA 355190)* | THX-U | HPC | 902493-06-5 | 247.70 | 3-^2^H,^13^C, 2-^15^N-ACE-N-DES |
| Nitenpyram | NIT | Chem Service | 120738-89-8 | 270.72 | NIT-d3 |
| *Nitenpyram-n-desmethyl* | NIT-N-DES | HPC | 120770-86-7 | 256.69 | 5-OH-IMI (2-^13^C, 3-^15^N, 2-amino-^15^N) |
| Flupyradifurone | FLU | Chem Service | 951659-40-8 | 288.68 | ACE-d3 |
| Flonicamid | FLO | Chem Service | 158062-67-0 | 229.16 | FLO-d3 |
| Sulfoxaflor | SUL | Chem Service | 946578-00-3 | 277.27 | ACE-d3 |
| *Sulfoxaflor metabolite X11719474* | SUL-X | HPC | 1186104-89-1 | 295.28 | CLO-N-DES (Guanidine-^13^C, Guanidine-1,3-^15^N_2_) |
| Imidaclothiz | IMZ | Santa Cruz Biotechnology | 105843-36-5 | 261.69 | IMZ-d4 |
| Acetamiprid-d3 | ACE-d3 | HPC | 1353869-35-8 | 225.69 | NA |
| Clothianidin-d3 | CLO-d3 | HPC | 1262776-24-8 | 252.70 | NA |
| Imidacloprid-d4 | IMI-d4 | HPC | 1015855-75-0 | 259.69 | NA |
| Thiacloprid-d4 | THC-d4 | HPC | 1793071-39-2 | 256.75 | NA |
| Thiamethoxam-d3 | THX-d3 | HPC | 1294048-82-0 | 294.73 | NA |
| Nitenpyram-d3 | NIT-d3 | CDN Isotopes | 150824-47-8 | 273.73 | NA |
| Flonicamid-d3 | FLO-d3 | HPC | NA | 229.16 | NA |
| Imidaclothiz-d4 | IMZ-d4 | TRC^*^ | NA | 265.71 | NA |
| 3-^2^H,^13^C, 2-^15^N-Acetamiprid-n-desmethyl | 3-^2^H,^13^C, 2-^15^N-ACE-N-DES | CanSyn Chemical | NA | 214 | NA |
| Clothianidin-n-desmethyl (Guanidine-^13^C, Guanidine-1,3-^15^N_2_) | CLO-N-DES (Guanidine-^13^C, Guanidine-1,3-^15^N_2_) | CIL^*^ | 2483830-32-4 | 238.62 | NA |
| 5-hydroxy-imidacloprid (2-^13^C, 3-^15^N, 2-amino-^15^N) | 5-OH-IMI (2-^13^C, 3-^15^N, 2-amino-^15^N) | CIL^*^ | NA | 274.64 | NA |
| Imidacloprid olefin (Imidazol-1-^15^N, 2-^13^C, 2-amino ^15^N) | IMI-O (Imidazol-1-^15^N, 2-^13^C, 2-amino ^15^N) | CIL^*^ | 2483830-31-3 | 256.62 | NA |
| Thiacloprid-amide (pyridylmethyl-^13^C_6_) | THC-A (pyridylmethyl-^13^C_6_) | CIL^*^ | 2483735-32-4 | 276.69 | NA |

^*^CIL: Cambridge Isotope Laboratories, Inc.

^*^TRC: Toronto Research Chemicals

**Table S2.** Preparation for calibrators, matrix spikes, matrix blank, and unknow samples. LRB: lab reagent blank.

| Std. | Vol. 100 ng/ml std. (uL) | Vol. 10 ng/ml std. (uL) | Vol. 1 ng/ml std. (uL) | Vol. 100 ng/ml ISTD (uL) | Urine (mL) | Vol. Milli-Q Water (mL) | Total Vol. Before Extraction (mL) | Total Vol. After Extraction (mL) |
| --- | --- | --- | --- | --- | --- | --- | --- | --- |
| 25 ng/ml cal | 250 |  |  | 50 |  |  | 1 | no extraction |
| 10 ng/ml cal | 100 |  |  | 50 |  |  | 1 |  |
| 5 ng/ml cal | 50 |  |  | 50 |  |  | 1 |  |
| 2.5 ng/ml cal | 25 |  |  | 50 |  |  | 1 |  |
| 2 ng/ml cal | 20 |  |  | 50 |  |  | 1 |  |
| 1.5 ng/ml cal | 15 |  |  | 50 |  |  | 1 |  |
| 1 ng/ml cal | 10 |  |  | 50 |  |  | 1 |  |
| 0.5 ng/ml cal |  | 50 |  | 50 |  |  | 1 |  |
| 0.25 ng/ml cal |  | 25 |  | 50 |  |  | 1 |  |
| 0.1 ng/ml cal |  | 10 |  | 50 |  |  | 1 |  |
| 0.05 ng/ml cal |  |  | 50 | 50 |  |  | 1 |  |
| 0.025 ng/ml cal |  |  | 25 | 50 |  |  | 1 |  |
| 0.01 ng/ml cal |  |  | 10 | 50 |  |  | 1 |  |
| 0 ng/ml (LRB) |  |  | 0 | 50 |  |  | 1 |  |
| QCH | 45 |  |  | 25 | 1 | 10 | 11 | 0.5 |
| QCMH | 15 |  |  | 25 | 1 | 10 | 11 | 0.5 |
| QCML |  | 25 |  | 25 | 1 | 10 | 11 | 0.5 |
| QCL |  |  |  | 25 | 1 | 10 | 11 | 0.5 |
| Matrix Blank |  |  |  | 25 | 1 | 10 | 11 | 0.5 |
| Unknow Sample |  |  |  | 25 | 1 | 10 | 11 | 0.5 |

**Table S3.** Procedures on Vivace^Tm^ Duo cleanup station. Solvent 1, 2, 3, and 4 is methanol, Milli-Q water, 20% methanol in Milli-Q water, and 0.5% formic acid in acetonitrile/ ethyl acetate (8:2 v: v), respectively. Vial 1 and 2 is samples tube and elution tube, respectively.

| No. | Action | Inlet | Target | Flow (mL/min) | Volume (mL) |
| --- | --- | --- | --- | --- | --- |
| 1 | Elute | Solvent 1 | Waste | 3 | 5.0 |
| 2 | Elute | Solvent 2 | Waste | 3 | 5.0 |
| 3 | Add Sample | Vial 1 | Waste | 2 | 15.0 |
| 4 | Clean | Solvent 2 | Vial 1 | 10 | 3.0 |
| 5 | Add Sample | Vial 1 | Waste | 2 | 5 |
| 6 | Air-Purge | Air | Waste | 5 | 5.0 |
| 7 | Elute | Solvent 3 | Waste | 2 | 3 |
| 8 | Air-Purge | Air | Waste | 5 | 5.0 |
| 9 | Blow N_2_ |  | Time based |  | 3 min |
| 10 | Collect | Solvent 4 | Vial 2 | 2 | 5 |
| 11 | Air-Purge | Air | Vial 2 | 5 | 5.0 |
| 14 | Wash Needle | Solvent 1 | Waste | 20 | 6 |


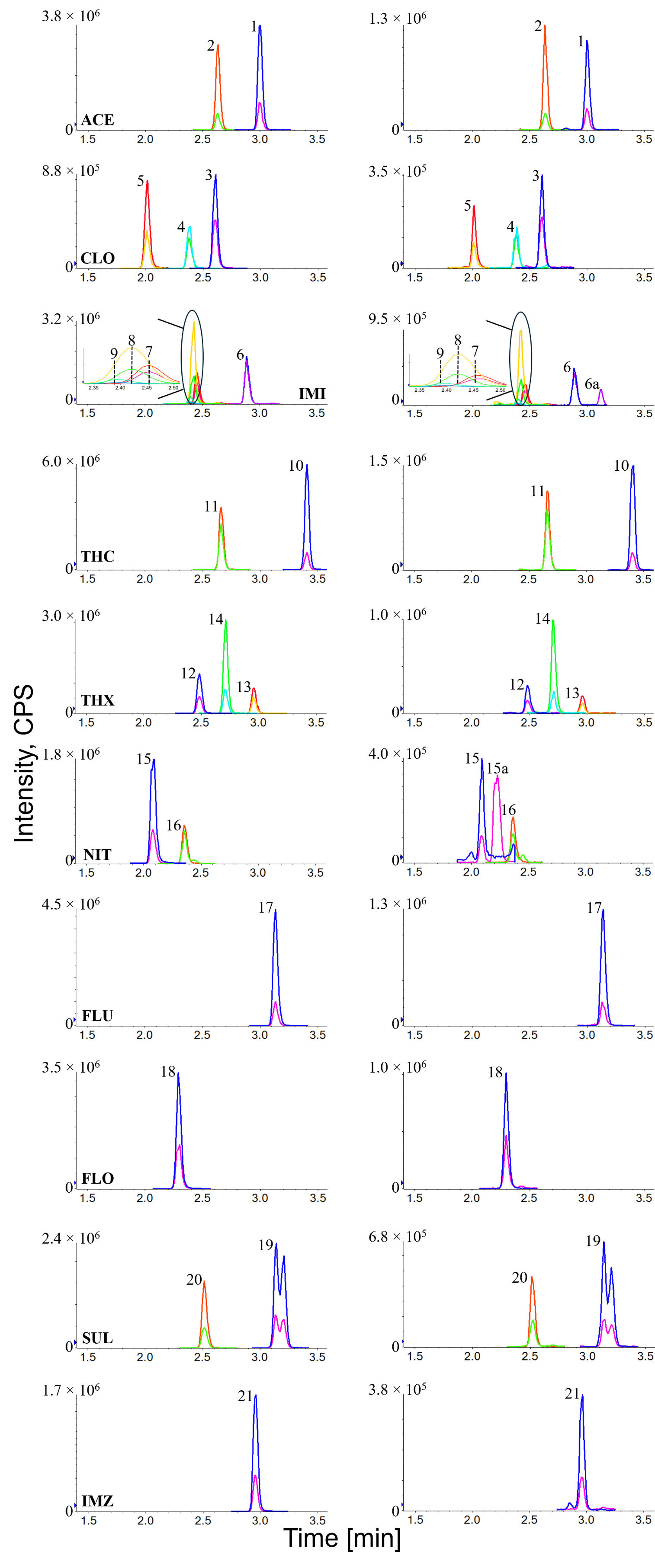


**Figure S1.** Extracted ion chromatograms of an aqueous calibration standard (3.5 ng/mL, left) and a quality control sample in pooled urine (3.5 ng/mL, right) for each NNI and its metabolites; the quantifier transitions of each analyte and confirmation ion transitions are shown (**1**: ACE, 2: ACE-N-DES, 3: CLO, 4: CLO-N-DES, 5: CLO-U, 6: IMI, 6a: unknown, 7: 5-OH-IMI, 8: IMI-U, 9: IMI-O, 10: THC, 11: THC-A, 12: THX, 13: THX-N-DES, 14: THX-U, 15: NIT, 15a: unknown, 16: NIT-N-DES, 17: FLU, 18: FLO, 19: SUL, 20: SUL-X, 21: IMZ.)

**Table S4.** Spearman Correlation Matrix for NEOs and NEO metabolites in urinary samples from Pregnant Women by year and date of analysis (n=246)

|  | **Batch Date** | **1-Year Period** | **2-Year Period** | **5-year Period** |
| --- | --- | --- | --- | --- |
| **ƩIMI** | 0.042 | -0.048 | -0.043 | -0.036 |
|  | 0.509 | 0.457 | 0.507 | 0.578 |
| **ƩCLO** | -0.099 | 0.106 | 0.104 | 0.099 |
|  | 0.120 | 0.098 | 0.104 | 0.123 |
| **ƩTHX** | -0.115 | **0.153** | **0.157** | **0.146** |
|  | 0.073 | **0.016** | **0.014** | **0.022** |
| **ƩACE** | -0.035 | **0.187** | **0.182** | **0.177** |
|  | 0.583 | **0.003** | **0.004** | **0.005** |
| **ACE** | -0.109 | -0.017 | -0.022 | -0.023 |
|  | 0.089 | 0.792 | 0.736 | 0.717 |
| **CLO** | **-0.178** | 0.096 | 0.096 | 0.080 |
|  | **0.005** | 0.135 | 0.133 | 0.209 |
| **IMI** | -0.021 | -0.037 | -0.035 | -0.064 |
|  | 0.744 | 0.565 | 0.590 | 0.316 |
| **THX** | -0.117 | **0.217** | **0.223** | **0.188** |
|  | 0.066 | **0.001** | **<.0001** | **0.003** |
| **NIT** | **-0.129** | 0.000 | 0.001 | -0.041 |
|  | **0.043** | 0.996 | 0.983 | 0.519 |
| **FLU** | -0.091 | **0.190** | **0.191** | **0.171** |
|  | 0.157 | **0.003** | **0.003** | **0.007** |
| **FLO** | -0.079 | 0.010 | 0.014 | -0.025 |
|  | 0.216 | 0.879 | 0.829 | 0.696 |
| **SUL** | **-0.231** | **0.200** | **0.195** | **0.165** |
|  | **<.0001** | **0.002** | **0.002** | **0.009** |
| **IMZ** | -0.064 | 0.023 | 0.027 | 0.009 |
|  | 0.315 | 0.715 | 0.676 | 0.889 |
| **ACE-N-DES** | -0.032 | **0.189** | **0.185** | **0.177** |
|  | 0.620 | **0.003** | **0.004** | **0.005** |
| **5-OH-IMI** | 0.045 | -0.059 | -0.046 | -0.061 |
|  | 0.484 | 0.360 | 0.469 | 0.338 |
| **THX-N-DES** | -0.084 | 0.057 | 0.060 | 0.045 |
|  | 0.188 | 0.373 | 0.350 | 0.482 |
| **CLO-U** | -0.038 | -0.002 | -0.001 | -0.005 |
|  | 0.558 | 0.979 | 0.990 | 0.936 |
| **THX-U** | 0.012 | 0.033 | 0.041 | 0.053 |
|  | 0.853 | 0.605 | 0.525 | 0.409 |
| **IMI-U** | -0.062 | 0.002 | 0.010 | -0.029 |
|  | 0.335 | 0.980 | 0.873 | 0.649 |
| **CLO-N-DES** | -0.039 | 0.064 | 0.062 | 0.075 |
|  | 0.548 | 0.318 | 0.334 | 0.243 |
| **SUL-X** | -0.106 | **0.151** | **0.152** | **0.129** |
|  | 0.096 | **0.018** | **0.017** | **0.044** |
| **IMI-O** | 0.046 | -0.073 | -0.073 | -0.049 |
|  | 0.474 | 0.257 | 0.257 | 0.444 |
| **ƩNEO** | -0.028 | **0.160** | **0.165** | **0.150** |
|  | 0.665 | **0.012** | **0.010** | **0.019** |
| **Detects / Sample** | **-0.192** | **0.182** | **0.181** | **0.145** |
|  | **0.002** | **0.004** | **0.004** | **0.023** |

**Table S5.** Kruskal-Wallis Analysis for NEOs and NEO metabolites in urinary samples from Pregnant Women by year.

|  | Year | | | 2-Year | | | 5-Year | | |  | Batch |  |
| --- | --- | --- | --- | --- | --- | --- | --- | --- | --- | --- | --- | --- |
|  | **Chi-Square** | **DF** | **Pr > ChiSq** | **Chi-Square** | **DF** | **Pr > ChiSq** | **Chi-Square** | **DF** | **Pr > ChiSq** | **Chi-Square** | **DF** | **Pr > ChiSq** |
| **ACE** | 13.068 | 15 | 0.597 | 7.760 | 7 | 0.354 | 0.956 | 2 | 0.620 | 14.380 | 10 | 0.156 |
| **CLO** | 14.766 | 15 | 0.468 | 8.206 | 7 | 0.315 | 4.101 | 2 | 0.129 | 11.914 | 10 | 0.291 |
| **IMI** | 24.571 | 15 | 0.056 | 6.060 | 7 | 0.533 | 1.189 | 2 | 0.552 | **36.377** | **10** | **<.0001** |
| **THX** | **26.742** | **15** | **0.031** | **22.479** | **7** | **0.002** | **8.734** | **2** | **0.013** | **19.078** | **10** | **0.039** |
| **NIT** | 19.419 | 15 | 0.195 | 11.377 | 7 | 0.123 | 3.578 | 2 | 0.167 | **19.156** | **10** | **0.038** |
| **FLU** | **25.753** | **15** | **0.041** | **15.887** | **7** | **0.026** | **10.519** | **2** | **0.005** | **27.680** | **10** | **0.002** |
| **FLO** | 14.297 | 15 | 0.503 | 5.784 | 7 | 0.565 | 0.758 | 2 | 0.685 | 18.188 | 10 | 0.052 |
| **SUL** | **38.553** | **15** | **0.001** | **29.832** | **7** | **<0.001** | **21.503** | **2** | **<.0001** | **35.229** | **10** | **<.0001** |
| **IMZ** | 14.216 | 15 | 0.509 | 3.575 | 7 | 0.827 | 1.167 | 2 | 0.558 | 18.074 | 10 | 0.054 |
| **ACE-N-DES** | **28.406** | **15** | **0.019** | **14.950** | **7** | **0.037** | **8.976** | **2** | **0.011** | 17.226 | 10 | 0.070 |
| **5-OH-IMI** | 16.117 | 15 | 0.374 | 9.192 | 7 | 0.239 | 1.345 | 2 | 0.510 | 16.430 | 10 | 0.088 |
| **THX-N-DES** | 15.996 | 15 | 0.382 | 4.331 | 7 | 0.741 | 2.256 | 2 | 0.324 | 18.021 | 10 | 0.055 |
| **CLO-U** | 11.525 | 15 | 0.715 | 5.215 | 7 | 0.634 | 0.106 | 2 | 0.948 | 15.481 | 10 | 0.116 |
| **THX-U** | 14.640 | 15 | 0.478 | 4.141 | 7 | 0.764 | 0.690 | 2 | 0.708 | 14.318 | 10 | 0.159 |
| **IMI-U** | 17.750 | 15 | 0.276 | 10.524 | 7 | 0.161 | 0.580 | 2 | 0.748 | 17.189 | 10 | 0.070 |
| **CLO-N-DES** | 7.609 | 15 | 0.939 | 3.846 | 7 | 0.797 | 1.702 | 2 | 0.427 | **18.603** | **10** | **0.046** |
| **SUL-X** | 22.357 | 15 | 0.099 | 12.356 | 7 | 0.090 | **9.337** | **2** | **0.009** | **32.710** | **10** | **<.0001** |
| **IMI-O** | 20.597 | 15 | 0.150 | 9.539 | 7 | 0.216 | 0.919 | 2 | 0.632 | 14.370 | 10 | 0.157 |
| **ƩNEO** | **30.653** | **15** | **0.010** | **14.456** | **7** | **0.044** | **6.463** | **2** | **0.040** | **20.040** | **10** | **0.029** |
| **Detects/Sample** | 22.754 | 15 | 0.090 | **17.078** | **7** | **0.017** | **6.130** | **2** | **0.047** | **21.452** | **10** | **0.018** |
| **ƩIMI** | 16.146 | 15 | 0.372 | 6.770 | 7 | 0.453 | 0.345 | 2 | 0.842 | 13.915 | 10 | 0.177 |
| **ƩCLO** | 10.273 | 15 | 0.802 | 7.081 | 7 | 0.421 | 2.838 | 2 | 0.242 | 12.281 | 10 | 0.267 |
| **ƩTHX** | 15.073 | 15 | 0.446 | 11.906 | 7 | 0.104 | **6.596** | **2** | **0.037** | **18.467** | **10** | **0.048** |
| **ƩACE** | **28.863** | **15** | **0.017** | **15.016** | **7** | **0.036** | **8.815** | **2** | **0.012** | 16.795 | 10 | 0.079 |

**Table S6.** Inter-day imprecision for NEOs and their metabolites (two months, n = 9) at three concentration level (QCL, QCM, and QCH). M_c_, mean concentration of analytes, RSD, relative standard deviation.

| **Analyte** | QCL | | QCM | | QCH | |
| --- | --- | --- | --- | --- | --- | --- |
|  | M_c_ | RSD | M_c_ | RSD | M_c_ | RSD |
| ACE | 0.38 | 7.1% | 1.84 | 10.2% | 5.23 | 3.9% |
| CLO | 0.67 | 6.1% | 2.13 | 10.0% | 5.54 | 5.0% |
| IMI | 0.40 | 7.6% | 1.82 | 8.4% | 5.19 | 5.2% |
| THC | 0.38 | 9.3% | 1.84 | 8.0% | 5.20 | 3.6% |
| THX | 0.51 | 9.8% | 1.96 | 10.4% | 5.70 | 2.4% |
| NIT | 0.39 | 10.8% | 1.69 | 6.9% | 4.71 | 6.1% |
| FLU | 0.40 | 12.4% | 1.80 | 9.3% | 4.86 | 11.2% |
| FLO | 0.41 | 5.9% | 1.91 | 9.5% | 5.43 | 4.9% |
| SUL | 0.52 | 15.5% | 2.39 | 16.8% | 6.36 | 7.7% |
| IMZ | 0.56 | 6.9% | 2.63 | 13.2% | 7.29 | 7.5% |
| ACE-N-DES | 0.47 | 11.9% | 1.94 | 9.1% | 5.10 | 7.5% |
| CLO-N-DES | 0.46 | 8.2% | 1.76 | 8.3% | 4.88 | 5.0% |
| CLO-U | 0.34 | 14.2% | 1.51 | 10.1% | 4.15 | 13.2% |
| 5-OH-IMI | 0.45 | 9.7% | 1.90 | 11.5% | 5.00 | 4.3% |
| IMI-U | 0.30 | 17.7% | 1.58 | 12.2% | 4.27 | 13.3% |
| IMI-O | 0.49 | 19.9% | 1.80 | 15.8% | 4.77 | 9.9% |
| THC-A | 0.45 | 10.2% | 2.17 | 12.5% | 6.22 | 6.8% |
| THX-N-DES | 0.39 | 7.3% | 1.86 | 13.5% | 5.05 | 6.9% |
| THX-U | 0.46 | 11.6% | 1.68 | 9.5% | 4.40 | 9.3% |
| NIT-N-DES | 0.36 | 13.3% | 1.84 | 15.7% | 4.94 | 12.7% |
| SUL-X | 0.56 | 15.9% | 2.32 | 16.2% | 6.61 | 12.3% |

As described in Section 2.4, QC materials prepared from pooled urine at three concentration level, QCL (~ 0.35 ng/mL), QCM (~ 1.75 ng/mL), and QCH (~ 4.75 ng/mL), were analyzed along with unknown samples. The results were monitored for two months and the mean concentration M_c_ and relative standard deviation RSD% were used to evaluate the inter-day imprecision (Table 2). All RSD results were below 20% indicates that the method is reliable.

**Table S7.** Spearman Correlation Matrix for NEOs and NEO metabolites in urinary samples from Pregnant Women, 2010-25 (n=246)

|  | **ACE** | **CLO** | **IMI** | **THX** | **NIT** | **FLU** | **FLO** | **SUL** | **IMZ** | **ACE-N-DES** | **5-OH-IMI** | **THX-N-DES** | **CLO-U** | **THX-U** | **IMI-U** | **CLO-N-DES** | **SUL-X** | **IMI-O** | **ƩNEO** | **Detects/ Sample** |
| --- | --- | --- | --- | --- | --- | --- | --- | --- | --- | --- | --- | --- | --- | --- | --- | --- | --- | --- | --- | --- |
| **ACE** | 1.000 | 0.252 | 0.454 | 0.264 | 0.733 | 0.725 | 0.749 | 0.532 | 0.838 | 0.243 | 0.205 | 0.779 | 0.769 | 0.598 | 0.749 | 0.150 | 0.631 | 0.167 | 0.313 | -0.024 |
|  |  | <.0001 | <.0001 | <.0001 | <.0001 | <.0001 | <.0001 | <.0001 | <.0001 | 0.000 | 0.001 | <.0001 | <.0001 | <.0001 | <.0001 | 0.018 | <.0001 | 0.009 | <.0001 | 0.711 |
| **CLO** | 0.252 | 1.000 | 0.247 | 0.388 | 0.196 | 0.288 | 0.173 | 0.354 | 0.214 | 0.257 | 0.260 | 0.223 | 0.141 | 0.166 | 0.178 | 0.489 | 0.302 | 0.278 | 0.485 | 0.543 |
|  | <.0001 |  | <.0001 | <.0001 | 0.002 | <.0001 | 0.006 | <.0001 | 0.001 | <.0001 | <.0001 | 0.000 | 0.027 | 0.009 | 0.005 | <.0001 | <.0001 | <.0001 | <.0001 | <.0001 |
| **IMI** | 0.454 | 0.247 | 1.000 | 0.252 | 0.461 | 0.452 | 0.482 | 0.219 | 0.505 | 0.254 | 0.616 | 0.424 | 0.454 | 0.354 | 0.462 | 0.270 | 0.369 | 0.526 | 0.556 | 0.352 |
|  | <.0001 | <.0001 |  | <.0001 | <.0001 | <.0001 | <.0001 | 0.001 | <.0001 | <.0001 | <.0001 | <.0001 | <.0001 | <.0001 | <.0001 | <.0001 | <.0001 | <.0001 | <.0001 | <.0001 |
| **THX** | 0.264 | 0.388 | 0.252 | 1.000 | 0.250 | 0.390 | 0.253 | 0.256 | 0.297 | 0.283 | 0.276 | 0.237 | 0.239 | 0.179 | 0.270 | 0.311 | 0.303 | 0.302 | 0.439 | 0.429 |
|  | <.0001 | <.0001 | <.0001 |  | <.0001 | <.0001 | <.0001 | <.0001 | <.0001 | <.0001 | <.0001 | <.0001 | <.0001 | 0.005 | <.0001 | <.0001 | <.0001 | <.0001 | <.0001 | <.0001 |
| **NIT** | 0.733 | 0.196 | 0.461 | 0.250 | 1.000 | 0.696 | 0.755 | 0.508 | 0.866 | 0.229 | 0.149 | 0.780 | 0.770 | 0.576 | 0.734 | 0.195 | 0.680 | 0.112 | 0.286 | -0.070 |
|  | <.0001 | 0.002 | <.0001 | <.0001 |  | <.0001 | <.0001 | <.0001 | <.0001 | <.0001 | 0.020 | <.0001 | <.0001 | <.0001 | <.0001 | 0.002 | <.0001 | 0.079 | <.0001 | 0.274 |
| **FLU** | 0.725 | 0.288 | 0.452 | 0.390 | 0.696 | 1.000 | 0.708 | 0.575 | 0.786 | 0.285 | 0.222 | 0.755 | 0.708 | 0.598 | 0.648 | 0.223 | 0.684 | 0.166 | 0.410 | 0.100 |
|  | <.0001 | <.0001 | <.0001 | <.0001 | <.0001 |  | <.0001 | <.0001 | <.0001 | <.0001 | 0.001 | <.0001 | <.0001 | <.0001 | <.0001 | <.0001 | <.0001 | 0.009 | <.0001 | 0.118 |
| **FLO** | 0.749 | 0.173 | 0.482 | 0.253 | 0.755 | 0.708 | 1.000 | 0.556 | 0.863 | 0.267 | 0.202 | 0.796 | 0.794 | 0.599 | 0.729 | 0.164 | 0.670 | 0.156 | 0.337 | -0.047 |
|  | <.0001 | 0.006 | <.0001 | <.0001 | <.0001 | <.0001 |  | <.0001 | <.0001 | <.0001 | 0.002 | <.0001 | <.0001 | <.0001 | <.0001 | 0.010 | <.0001 | 0.014 | <.0001 | 0.468 |
| **SUL** | 0.532 | 0.354 | 0.219 | 0.256 | 0.508 | 0.575 | 0.556 | 1.000 | 0.601 | 0.309 | 0.134 | 0.632 | 0.532 | 0.455 | 0.466 | 0.275 | 0.653 | 0.139 | 0.340 | 0.168 |
|  | <.0001 | <.0001 | 0.001 | <.0001 | <.0001 | <.0001 | <.0001 |  | <.0001 | <.0001 | 0.036 | <.0001 | <.0001 | <.0001 | <.0001 | <.0001 | <.0001 | 0.029 | <.0001 | 0.008 |
| **IMZ** | 0.838 | 0.214 | 0.505 | 0.297 | 0.866 | 0.786 | 0.863 | 0.601 | 1.000 | 0.255 | 0.205 | 0.906 | 0.895 | 0.703 | 0.823 | 0.225 | 0.769 | 0.172 | 0.328 | -0.117 |
|  | <.0001 | 0.001 | <.0001 | <.0001 | <.0001 | <.0001 | <.0001 | <.0001 |  | <.0001 | 0.001 | <.0001 | <.0001 | <.0001 | <.0001 | <.0001 | <.0001 | 0.007 | <.0001 | 0.067 |
| **ACE-N-DES** | 0.243 | 0.257 | 0.254 | 0.283 | 0.229 | 0.285 | 0.267 | 0.309 | 0.255 | 1.000 | 0.311 | 0.213 | 0.254 | 0.205 | 0.199 | 0.221 | 0.293 | 0.300 | 0.606 | 0.374 |
|  | <.0001 | <.0001 | <.0001 | <.0001 | <.0001 | <.0001 | <.0001 | <.0001 | <.0001 |  | <.0001 | 0.001 | <.0001 | 0.001 | 0.002 | 0.001 | <.0001 | <.0001 | <.0001 | <.0001 |
| **5-OH-IMI** | 0.205 | 0.260 | 0.616 | 0.276 | 0.149 | 0.222 | 0.202 | 0.134 | 0.205 | 0.311 | 1.000 | 0.176 | 0.204 | 0.184 | 0.213 | 0.174 | 0.231 | 0.698 | 0.702 | 0.510 |
|  | 0.001 | <.0001 | <.0001 | <.0001 | 0.020 | 0.001 | 0.002 | 0.036 | 0.001 | <.0001 |  | 0.006 | 0.001 | 0.004 | 0.001 | 0.006 | <.0001 | <.0001 | <.0001 | <.0001 |
| **THX-N-DES** | 0.779 | 0.223 | 0.424 | 0.237 | 0.780 | 0.755 | 0.796 | 0.632 | 0.906 | 0.213 | 0.176 | 1.000 | 0.837 | 0.672 | 0.765 | 0.203 | 0.740 | 0.110 | 0.274 | -0.113 |
|  | <.0001 | <.0001 | <.0001 | <.0001 | <.0001 | <.0001 | <.0001 | <.0001 | <.0001 | 0.001 | 0.006 |  | <.0001 | <.0001 | <.0001 | 0.001 | <.0001 | 0.085 | <.0001 | 0.078 |
| **CLO-U** | 0.769 | 0.141 | 0.454 | 0.239 | 0.770 | 0.708 | 0.794 | 0.532 | 0.895 | 0.254 | 0.204 | 0.837 | 1.000 | 0.670 | 0.791 | 0.219 | 0.671 | 0.159 | 0.298 | -0.113 |
|  | <.0001 | 0.027 | <.0001 | <.0001 | <.0001 | <.0001 | <.0001 | <.0001 | <.0001 | <.0001 | 0.001 | <.0001 |  | <.0001 | <.0001 | 0.001 | <.0001 | 0.012 | <.0001 | 0.077 |
| **THX-U** | 0.598 | 0.166 | 0.354 | 0.179 | 0.576 | 0.598 | 0.599 | 0.455 | 0.703 | 0.205 | 0.184 | 0.672 | 0.670 | 1.000 | 0.591 | 0.209 | 0.621 | 0.168 | 0.282 | -0.023 |
|  | <.0001 | 0.009 | <.0001 | 0.005 | <.0001 | <.0001 | <.0001 | <.0001 | <.0001 | 0.001 | 0.004 | <.0001 | <.0001 |  | <.0001 | 0.001 | <.0001 | 0.008 | <.0001 | 0.724 |
| **IMI-U** | 0.749 | 0.178 | 0.462 | 0.270 | 0.734 | 0.648 | 0.729 | 0.466 | 0.823 | 0.199 | 0.213 | 0.765 | 0.791 | 0.591 | 1.000 | 0.138 | 0.616 | 0.149 | 0.286 | -0.031 |
|  | <.0001 | 0.005 | <.0001 | <.0001 | <.0001 | <.0001 | <.0001 | <.0001 | <.0001 | 0.002 | 0.001 | <.0001 | <.0001 | <.0001 |  | 0.030 | <.0001 | 0.019 | <.0001 | 0.628 |
| **CLO-N-DES** | 0.150 | 0.489 | 0.270 | 0.311 | 0.195 | 0.223 | 0.164 | 0.275 | 0.225 | 0.221 | 0.174 | 0.203 | 0.219 | 0.209 | 0.138 | 1.000 | 0.179 | 0.228 | 0.394 | 0.421 |
|  | 0.018 | <.0001 | <.0001 | <.0001 | 0.002 | <.0001 | 0.010 | <.0001 | <.0001 | 0.001 | 0.006 | 0.001 | 0.001 | 0.001 | 0.030 |  | 0.005 | <.0001 | <.0001 | <.0001 |
| **SUL-X** | 0.631 | 0.302 | 0.369 | 0.303 | 0.680 | 0.684 | 0.670 | 0.653 | 0.769 | 0.293 | 0.231 | 0.740 | 0.671 | 0.621 | 0.616 | 0.179 | 1.000 | 0.211 | 0.397 | 0.103 |
|  | <.0001 | <.0001 | <.0001 | <.0001 | <.0001 | <.0001 | <.0001 | <.0001 | <.0001 | <.0001 | <.0001 | <.0001 | <.0001 | <.0001 | <.0001 | 0.005 |  | 0.001 | <.0001 | 0.106 |
| **IMI-O** | 0.167 | 0.278 | 0.526 | 0.302 | 0.112 | 0.166 | 0.156 | 0.139 | 0.172 | 0.300 | 0.698 | 0.110 | 0.159 | 0.168 | 0.149 | 0.228 | 0.211 | 1.000 | 0.735 | 0.431 |
|  | 0.009 | <.0001 | <.0001 | <.0001 | 0.079 | 0.009 | 0.014 | 0.029 | 0.007 | <.0001 | <.0001 | 0.085 | 0.012 | 0.008 | 0.019 | <.0001 | 0.001 |  | <.0001 | <.0001 |
| **ƩNEO** | 0.313 | 0.485 | 0.556 | 0.439 | 0.286 | 0.410 | 0.337 | 0.340 | 0.328 | 0.606 | 0.702 | 0.274 | 0.298 | 0.282 | 0.286 | 0.394 | 0.397 | 0.735 | 1.000 | 0.569 |
|  | <.0001 | <.0001 | <.0001 | <.0001 | <.0001 | <.0001 | <.0001 | <.0001 | <.0001 | <.0001 | <.0001 | <.0001 | <.0001 | <.0001 | <.0001 | <.0001 | <.0001 | <.0001 |  | <.0001 |
| **Detects / Sample** | -0.024 | 0.543 | 0.352 | 0.429 | -0.070 | 0.100 | -0.047 | 0.168 | -0.117 | 0.374 | 0.510 | -0.113 | -0.113 | -0.023 | -0.031 | 0.421 | 0.103 | 0.431 | 0.569 | 1.000 |
|  | 0.711 | <.0001 | <.0001 | <.0001 | 0.274 | 0.118 | 0.468 | 0.008 | 0.067 | <.0001 | <.0001 | 0.078 | 0.077 | 0.724 | 0.628 | <.0001 | 0.106 | <.0001 | <.0001 |  |

**Table S8.** Spearman Correlation Matrix for NEOs and NEO metabolites in urinary samples from farmers, 2018-19 (n=47)

|  | **ACE** | **CLO** | **IMI** | **THX** | **NIT** | **FLU** | **FLO** | **SUL** | **IMZ** | **ACE-N-DES** | **5-OH-IMI** | **THX-N-DES** | **THX-U** | **CLO-N-DES** | **IMI-O** | **ƩNEO** | **Detects / Sample** |
| --- | --- | --- | --- | --- | --- | --- | --- | --- | --- | --- | --- | --- | --- | --- | --- | --- | --- |
| **ACE** | 1.000 | 0.196 | 0.107 | 0.397 | 0.210 | 0.754 | 0.428 | 0.569 | 0.023 | 0.310 | 0.090 | 0.119 | 0.510 | 0.407 | 0.252 | 0.208 | -0.075 |
|  |  | 0.186 | 0.472 | 0.006 | 0.157 | <.0001 | 0.003 | <.0001 | 0.879 | 0.034 | 0.548 | 0.426 | <.0001 | 0.005 | 0.087 | 0.160 | 0.617 |
| **CLO** | 0.196 | 1.000 | -0.025 | 0.582 | 0.177 | 0.329 | 0.039 | 0.183 | 0.269 | 0.315 | 0.214 | 0.240 | 0.272 | 0.642 | 0.262 | 0.456 | 0.445 |
|  | 0.186 |  | 0.865 | <.0001 | 0.234 | 0.024 | 0.793 | 0.219 | 0.068 | 0.031 | 0.149 | 0.104 | 0.065 | <.0001 | 0.075 | 0.001 | 0.002 |
| **IMI** | 0.107 | -0.025 | 1.000 | 0.063 | 0.325 | -0.091 | 0.076 | 0.134 | 0.297 | -0.148 | -0.082 | 0.425 | -0.051 | -0.158 | 0.107 | 0.494 | -0.169 |
|  | 0.472 | 0.865 |  | 0.674 | 0.026 | 0.542 | 0.613 | 0.369 | 0.042 | 0.321 | 0.582 | 0.003 | 0.734 | 0.290 | 0.474 | <.0001 | 0.257 |
| **THX** | 0.397 | 0.582 | 0.063 | 1.000 | 0.267 | 0.326 | 0.162 | 0.179 | 0.480 | 0.292 | 0.022 | 0.338 | 0.299 | 0.431 | 0.189 | 0.472 | 0.206 |
|  | 0.006 | <.0001 | 0.674 |  | 0.069 | 0.025 | 0.278 | 0.230 | 0.001 | 0.046 | 0.885 | 0.020 | 0.041 | 0.003 | 0.204 | 0.001 | 0.165 |
| **NIT** | 0.210 | 0.177 | 0.325 | 0.267 | 1.000 | -0.079 | -0.012 | 0.160 | 0.464 | 0.260 | -0.155 | 0.528 | -0.083 | -0.013 | 0.029 | 0.562 | -0.114 |
|  | 0.157 | 0.234 | 0.026 | 0.069 |  | 0.596 | 0.937 | 0.284 | 0.001 | 0.077 | 0.297 | <.0001 | 0.579 | 0.932 | 0.846 | <.0001 | 0.444 |
| **FLU** | 0.754 | 0.329 | -0.091 | 0.326 | -0.079 | 1.000 | 0.405 | 0.548 | -0.122 | 0.403 | 0.287 | -0.030 | 0.759 | 0.659 | 0.405 | 0.077 | 0.102 |
|  | <.0001 | 0.024 | 0.542 | 0.025 | 0.596 |  | 0.005 | <.0001 | 0.412 | 0.005 | 0.050 | 0.843 | <.0001 | <.0001 | 0.005 | 0.607 | 0.497 |
| **FLO** | 0.428 | 0.039 | 0.076 | 0.162 | -0.012 | 0.405 | 1.000 | 0.256 | -0.239 | 0.289 | 0.108 | -0.035 | 0.123 | 0.205 | 0.102 | 0.052 | -0.124 |
|  | 0.003 | 0.793 | 0.613 | 0.278 | 0.937 | 0.005 |  | 0.082 | 0.105 | 0.049 | 0.471 | 0.815 | 0.410 | 0.167 | 0.497 | 0.730 | 0.406 |
| **SUL** | 0.569 | 0.183 | 0.134 | 0.179 | 0.160 | 0.548 | 0.256 | 1.000 | -0.023 | 0.403 | 0.281 | 0.197 | 0.418 | 0.271 | 0.482 | 0.232 | 0.090 |
|  | <.0001 | 0.219 | 0.369 | 0.230 | 0.284 | <.0001 | 0.082 |  | 0.876 | 0.005 | 0.055 | 0.185 | 0.003 | 0.066 | 0.001 | 0.117 | 0.548 |
| **IMZ** | 0.023 | 0.269 | 0.297 | 0.480 | 0.464 | -0.122 | -0.239 | -0.023 | 1.000 | 0.003 | -0.221 | 0.743 | 0.044 | -0.049 | 0.002 | 0.518 | 0.056 |
|  | 0.879 | 0.068 | 0.042 | 0.001 | 0.001 | 0.412 | 0.105 | 0.876 |  | 0.982 | 0.136 | <.0001 | 0.770 | 0.744 | 0.989 | <.0001 | 0.710 |
| **ACE-N-DES** | 0.310 | 0.315 | -0.148 | 0.292 | 0.260 | 0.403 | 0.289 | 0.403 | 0.003 | 1.000 | 0.282 | 0.153 | 0.400 | 0.486 | 0.338 | 0.388 | 0.363 |
|  | 0.034 | 0.031 | 0.321 | 0.046 | 0.077 | 0.005 | 0.049 | 0.005 | 0.982 |  | 0.055 | 0.306 | 0.005 | 0.001 | 0.020 | 0.007 | 0.012 |
| **5-OH-IMI** | 0.090 | 0.214 | -0.082 | 0.022 | -0.155 | 0.287 | 0.108 | 0.281 | -0.221 | 0.282 | 1.000 | 0.027 | 0.242 | 0.279 | 0.591 | 0.197 | 0.730 |
|  | 0.548 | 0.149 | 0.582 | 0.885 | 0.297 | 0.050 | 0.471 | 0.055 | 0.136 | 0.055 |  | 0.859 | 0.101 | 0.058 | <.0001 | 0.184 | <.0001 |
| **THX-N-DES** | 0.119 | 0.240 | 0.425 | 0.338 | 0.528 | -0.030 | -0.035 | 0.197 | 0.743 | 0.153 | 0.027 | 1.000 | 0.079 | 0.051 | 0.184 | 0.652 | 0.151 |
|  | 0.426 | 0.104 | 0.003 | 0.020 | <.0001 | 0.843 | 0.815 | 0.185 | <.0001 | 0.306 | 0.859 |  | 0.600 | 0.732 | 0.217 | <.0001 | 0.310 |
| **THX-U** | 0.510 | 0.272 | -0.051 | 0.299 | -0.083 | 0.759 | 0.123 | 0.418 | 0.044 | 0.400 | 0.242 | 0.079 | 1.000 | 0.477 | 0.269 | 0.167 | 0.301 |
|  | <.0001 | 0.065 | 0.734 | 0.041 | 0.579 | <.0001 | 0.410 | 0.003 | 0.770 | 0.005 | 0.101 | 0.600 |  | 0.001 | 0.067 | 0.263 | 0.040 |
| **CLO-N-DES** | 0.407 | 0.642 | -0.158 | 0.431 | -0.013 | 0.659 | 0.205 | 0.271 | -0.049 | 0.486 | 0.279 | 0.051 | 0.477 | 1.000 | 0.396 | 0.236 | 0.468 |
|  | 0.005 | <.0001 | 0.290 | 0.003 | 0.932 | <.0001 | 0.167 | 0.066 | 0.744 | 0.001 | 0.058 | 0.732 | 0.001 |  | 0.006 | 0.110 | 0.001 |
| **IMI-O** | 0.252 | 0.262 | 0.107 | 0.189 | 0.029 | 0.405 | 0.102 | 0.482 | 0.002 | 0.338 | 0.591 | 0.184 | 0.269 | 0.396 | 1.000 | 0.449 | 0.369 |
|  | 0.087 | 0.075 | 0.474 | 0.204 | 0.846 | 0.005 | 0.497 | 0.001 | 0.989 | 0.020 | <.0001 | 0.217 | 0.067 | 0.006 |  | 0.002 | 0.011 |
| **ƩNEO** | 0.208 | 0.456 | 0.494 | 0.472 | 0.562 | 0.077 | 0.052 | 0.232 | 0.518 | 0.388 | 0.197 | 0.652 | 0.167 | 0.236 | 0.449 | 1.000 | 0.270 |
|  | 0.160 | 0.001 | <.0001 | 0.001 | <.0001 | 0.607 | 0.730 | 0.117 | <.0001 | 0.007 | 0.184 | <.0001 | 0.263 | 0.110 | 0.002 |  | 0.067 |
| **Detects / Sample** | -0.075 | 0.445 | -0.169 | 0.206 | -0.114 | 0.102 | -0.124 | 0.090 | 0.056 | 0.363 | 0.730 | 0.151 | 0.301 | 0.468 | 0.369 | 0.270 | 1.000 |
|  | 0.617 | 0.002 | 0.257 | 0.165 | 0.444 | 0.497 | 0.406 | 0.548 | 0.710 | 0.012 | <.0001 | 0.310 | 0.040 | 0.001 | 0.011 | 0.067 |  |
